# Chemical contaminant levels in edible seaweeds of the Salish Sea and implications for their consumption

**DOI:** 10.1371/journal.pone.0269269

**Published:** 2022-09-23

**Authors:** Jennifer L. Hahn, Kathryn L. Van Alstyne, Joseph K. Gaydos, Lindsay K. Wallis, James E. West, Steven J. Hollenhorst, Gina M. Ylitalo, Robert H. Poppenga, Jennie L. Bolton, David E. McBride, Ruth M. Sofield

**Affiliations:** 1 Department of Environmental Studies, Western Washington University, Bellingham, Washington, United States of America; 2 Shannon Point Marine Center, Western Washington University, Anacortes, Washington, United States of America; 3 The SeaDoc Society, Karen C. Drayer Wildlife Health Center - Orcas Island Office, University of California Davis, Eastsound, Washington, United States of America; 4 Department of Environmental Sciences, Western Washington University, Bellingham, Washington, United States of America; 5 Washington State Department of Fish and Wildlife, Olympia, Washington, United States of America; 6 Department of Urban and Environmental Planning and Policy, Western Washington University, Bellingham, Washington, United States of America; 7 Northwest Fisheries Science Center, National Marine Fisheries Service, Seattle, Washington, United States of America; 8 California Animal Health and Food Safety Laboratory System, Davis Branch, School of Veterinary Medicine, University of California Davis, Davis, California, United States of America; 9 Washington Department of Health Office of Environmental Health Assessments Olympia, Washington, United States of America; Zagazig University, EGYPT

## Abstract

Despite growing interest in edible seaweeds, there is limited information on seaweed chemical contaminant levels in the Salish Sea. Without this knowledge, health-based consumption advisories can not be determined for consumers that include Tribes and First Nations, Asian and Pacific Islander community members, and recreational harvesters. We measured contaminant concentrations in edible seaweeds (*Fucus distichus*, *F*. *spiralis*, and *Nereocystis luetkeana*) from 43 locations in the Salish Sea. Metals were analyzed in all samples, and 94 persistent organic pollutants (POPs) (i.e. 40 PCBs, 15 PBDEs, 17 PCDD/Fs, and 22 organochlorine pesticides) and 51 PAHs were analyzed in *Fucus* spp. We compared concentrations of contaminants to human health-based screening levels calculated from the USEPA and to international limits. We then worked with six focal contaminants that either exceeded screening levels or international limits (Cd, total Hg, Pb, benzo[*a*]pyrene [BaP], and PCBs) or are of regional interest (total As). USEPA cancer-based screening levels were exceeded in 30 samples for the PCBs and two samples for BaP. Cadmium concentrations did not exceed the USEPA noncancer-based screening level but did exceed international limits at all sites. Lead exceeded international limits at three sites. Because there are no screening levels for total Hg and total As, and to be conservative, we made comparisons to methyl Hg and inorganic As screening levels. All samples were below the methyl Hg and above the inorganic As screening levels. Without knowledge of the As speciation, we cannot assess the health risk associated with the As. While seaweed was the focus, we did not consider contaminant exposure from consuming other foods. Other chemicals, such as contaminants of emerging concern (e.g., PFAS, pharmaceuticals and personal care products), should also be considered. Additionally, although we focused on toxicological aspects, there are cultural and health benefits of seaweed use that may affect consumer choice.

## 1.0 Introduction

Seaweed (macroalgae) consumption is increasing globally [1]. The annual estimated growth rate of the worldwide seaweed market is 8–10% and the global annual harvest of seaweed is 36 million metric tons (about 80 billion pounds) and valued at approximately $6 billion USD for all seaweed species and seaweed-based products [2]. Western use of seaweed has historically focused on extracting algal phycocolloids (e.g., agar, carrageenan and alginate) for thickeners and emulsifiers [3], but consumption of whole seaweeds and seaweed-based products is increasing [4]. Seaweeds are generally eaten as a condiment, salt substitute, noodle, or as ingredients in sushi rolls, soups, salads (e.g., sunomono), fermented dishes (e.g., kimchi), and stir-fries [3]. They are also marketed commercially for snacks, teas, and meat substitutes. Major factors driving the interest in edible seaweed are their nutritional and health benefits, which potentially include the prevention of Type II diabetes, Alzheimer’s disease, cardiovascular disease, obesity, cancer, high blood pressure, and depression [5, 6], as well as a growing global market for nutraceuticals and for sustainable, plant-based food products with umami flavor [1, 7].

While seaweeds can absorb beneficial vitamins and minerals from sediments and seawater [8], they can also take up and concentrate organic and inorganic chemical contaminants [8–10]. Seaweeds are known to accumulate toxic substances such as lead (Pb), mercury (Hg), cadmium (Cd), arsenic (As), polycyclic aromatic hydrocarbons (PAHs), polychlorinated biphenyls (PCBs), polychlorinated-dibenzo-*p*-dioxins (PCDDs), polychlorinated-dibenzofurans (PCDFs), polybrominated diphenyl ethers (PBDEs), and organochlorine pesticides such as DDTs. These contaminants pose a risk to consumers if they are present in sufficient amounts. At this point, there are many gaps in our ability to assess the health risks that contaminants in seaweeds pose, including limited knowledge of their concentrations [9] and geographic distributions.

Regulations on allowable levels of contaminants for edible seaweeds and other seaweed-based products vary by country or region (Table 1). There are no regulations or maximum levels of contaminants in seaweed for human consumption in the US or Canada; there are limited regulations in Europe, where seaweeds can be considered and regulated as novel foods. In France, where 21 macroalgae and 3 microalgae species are authorized as condiments and vegetables, there are maximum levels allowed in all edible seaweeds for Pb, Cd, Hg, and inorganic As. China, Australia, New Zealand, and Mauritius also have regulated levels for some metals in seaweeds sold as foods (Table 1). The European Commission assigned a Cd limit (3.0 mg/kg WW) for food supplements consisting exclusively or mainly of dried seaweed and products derived from seaweed. These maximum levels for food supplements are for the product as sold [18] (Table 1).

**Table 1:** Contaminant regulations and limits in seaweed.

Analyte	Country	Amount	Comments
Cadmium	Australia/New Zealand	0.2 mg/kg DW ^i^	-----
Cadmium	France	0.5 mg/kg DW ^d^ ^(cited in^ ^b^^),^ ^e^^,^ ^k^	-----
Cadmium	China	1 mg/kg ^j^ ^cited in^ ^m^	Appears to be based on DW, but this could not be verified
Cadmium	European Commission	3 mg/kg WW^h^	Applies to supplements containing exclusively or mainly dried seaweeds
Mercury	France	0.1 mg/kg DW ^d^ ^(cited in^ ^b^^),^ ^e^^,^ ^k^	Type of Hg is not specified
Mercury	European Commission	0.10 mg/kg KK ^h^	In food supplements
Inorganic Arsenic	Australia/New Zealand	6.7 mg/kg DW ^c^	Calculated from limit of 1 mg/kg WW at 85% hydration
Inorganic Arsenic	China	1 mg/kg ^i^ ^(cited in^ ^m^^)^	Appears to be based on DW, but this could not be verified; the standard for “free-pollutant” food according to Zhao et al. (2012)
Inorganic Arsenic	China	1.5 mg/kg ^l^ ^(cited in^ ^m^^)^	Appears to be based on DW, but this could not be verified
Inorganic Arsenic	France	3 mg/kg DW ^a^	-----
Lead	China	1 mg/kg DW ^f^	-----
Lead	France	5 mg/kg DW ^d^ ^(cited in^ ^k^^)^	-----
Lead	Mauritius	10 mg/kg ^7^	WW or DW not specified
Lead	European Commission	3.0 mg/kg WW^h^	In food supplements

Representative upper limits or maximum levels of the amounts of cadmium, arsenic, lead, and mercury in regulations or recommendations concerning seaweeds for human consumption. DW: dry weight, WW: wet weight.

^a^[11],

^b^[12],

^c^[13],

^d^[14],

^e^[15],

^f^[16],

^g^[17],

^h^[18],

^i^[19],

^j^[20],

^k^[21],

^l^[22],

^m^[23]

There is increased interest in consuming farmed and wild-harvested seaweeds in the Salish Sea in the western United States and Canada where, in addition to nutritional and health benefits, cultural practices and small-business interests drive seaweed consumption. Salish Sea seaweed consumers include Indigenous people using traditional food sources [24, 25]; recreational harvesters [26]; Asian and Pacific Islander community members using traditional foodways [27]; chefs and high-end restaurants seeking novel textures and flavors [28, 29]; and gardeners who seek mulch and soil amendments [30]. Regional online companies now sell Salish Sea seaweeds as value-added products such as snacks, teas, seasonings, condiments, skincare products, and distillery spirits. In Washington state’s Hood Canal area, kelp is cultivated to provide soil amendments for farming vegetables and a potential source of food with the added benefit of mitigating ocean acidification [31]. Although levels of organic and inorganic chemicals in Salish Sea sediment and marine animals are routinely investigated [e.g., 32–36], only a handful of studies have investigated contaminants in Salish Sea seaweeds; most of these were limited in geographic area or number of contaminants analyzed and were conducted over two decades ago [37–39].

To better understand the potential impacts of contaminants in edible seaweeds over a large geographic area, we measured concentrations of 162 contaminants in three commonly consumed species of brown seaweeds at 43 unique sites within the Salish Sea. We compared these concentrations to risk-based screening levels (SLs) based on reference doses (RfDs) and cancer screening factors (CSFs), which is the approach used by the USEPA for fish consumption advisories (USEPA, 2000). This approach is used in the US to help agencies determine whether fish consumers are potentially overexposed to levels of contaminants that exceed health benchmarks related to cancer risks and potential non-cancer health hazards, and to develop meal recommendations for people consuming these fish; the approach is also used for shellfish and can be applied to seaweed. RfDs and CSFs estimate the daily quantity of a contaminant that a population can be exposed to without significant risks of non-carcinogenic (RfD) [40] and carcinogenic (CSF) health effects over a lifetime [41]. We also compared our seaweed contaminant concentrations to international levels when they were available. Based on either contaminants exceeding screening levels or international limits (PCBs, benzo[*a*]pyrene [BaP], Cd, total Hg, and Pb) and concern about As concentrations in Salish Sea seafoods (total As), we then compared levels of these six contaminants (hereafter referred to as focal contaminants) to their concentrations in commonly eaten foods. We also calculated site-specific, species-based recommendations for consumption rates.

## 2.0 Methods

### 2.1 Study species and area

*Fucus distichus* and *Fucus spiralis* (also known as rockweed or bladderwrack) are commonly found in the intertidal zone of rocky shores of the Salish Sea, a large (approximately 18,000 km^2^) international inland sea whose watersheds drain a wide range of land-use types including highly urbanized areas, residential developments, agricultural areas, and relatively pristine habitats. Harvesters in Washington (WA) State and in British Columbia (BC), gather both vegetative blades and reproductive receptacles from *Fucus* for use in foods, teas, and medicines [42]. Bull or bull whip kelp *Nereocystis luetkeana* is a subtidal brown alga that is common in nearshore habitats. Both blades and stipes are harvested for food, medicine, agriculture, and mariculture [42, 43].

**Fig 1:**
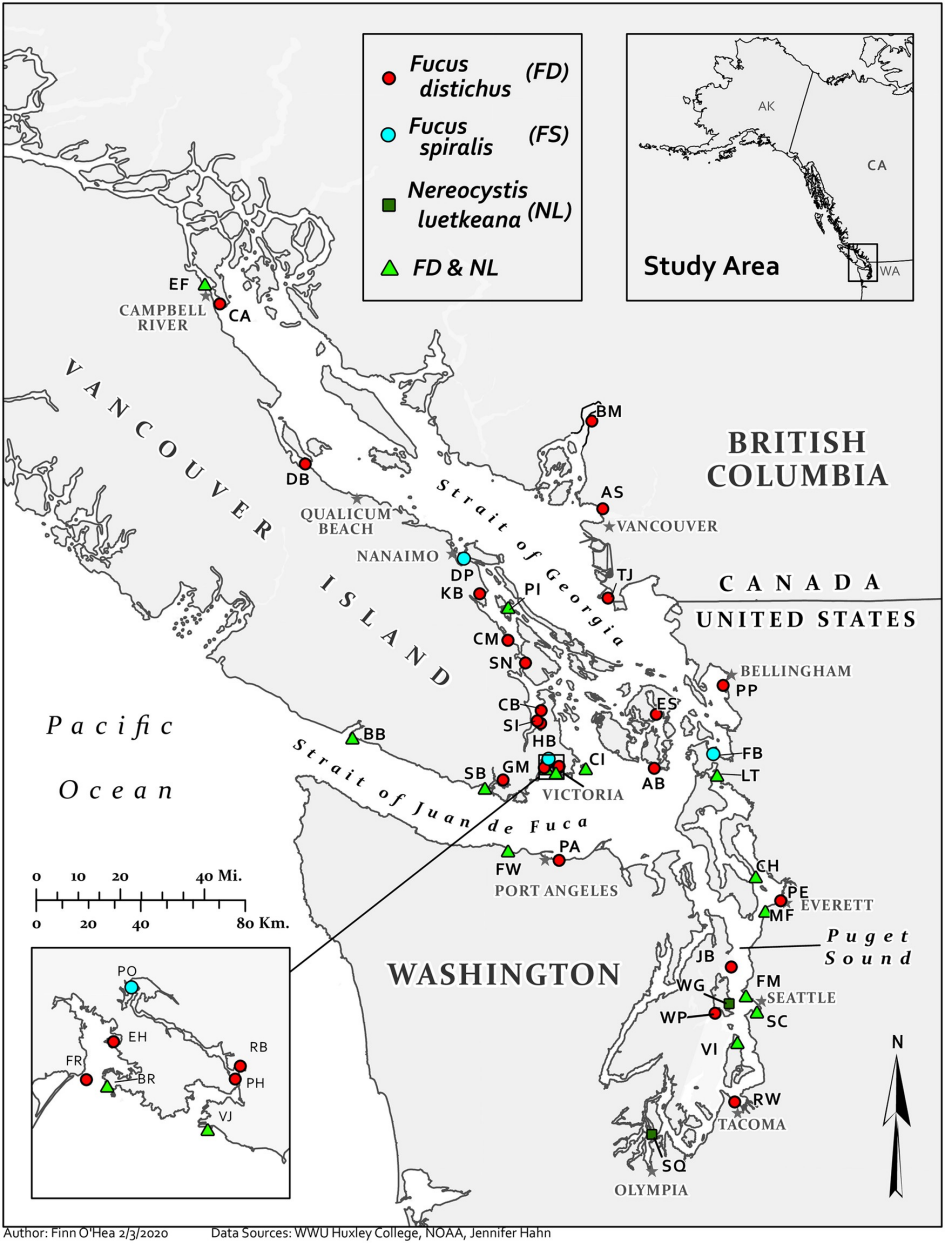
Sites sampled in the Salish Sea. AB: Agate Beach, AS: Ambleside, BB: Botanical Beach, BM: Britannia Mine, BR: Brothers Islands/Duntze Head, CA: Cape Mudge, CB: Coles Bay, CH: Camano Head, CI: Chatham Islands/*Tl’chés*, CM: Crofton Mill, DB: Deep Bay, DP: Duke Point Industrial Park, EF: Elk Falls Pulp Mill, CR: Campbell River, EH: Esquimalt Harbour, ES: East Sound, FW: Fresh Water Bay, FB: Fidalgo Bay Aquatic Reserve, FM: Four-Mile Rock, FR: Fort Rod Hill, GM: Goodridge Mill, PO: Portage Inlet HB: Hagan Bight, JB: Jefferson Beach, KB: Kulleet Bay, LT: Lone Tree Point, MF: Mukilteo Ferry, PA: Port Angeles, PE: Port of Everett, PH: Point Hope Shipyard, PI: Penelakut Island, PP: Post Point, PO: Portage Inlet, RB: Rock Bay, RW: Ruston Way, SB: Sooke Bay, SC: Smith Cove, SI: Senanus Island, SN: Sansum Narrows, SQ: Squaxin Island, TJ: Tsawwassen Jetty, VI: Vashon Island, VJ: Victoria Jetty, WP: Waterman Point, WG: Wing Point. Red circles indicate sites where only *F*. *distichu*s were sampled, blue circles indicate sites where only *F*. *spiralis* were sampled, green squares indicate sites where only *N*. *luetkeana* were sampled, and green triangles indicate sites where both *F*. *distichus* and *N*. *luetkeana* were sampled.

Intertidal *Fucus distichus* from 38 sites, intertidal *Fucus spiralis* from three sites, and subtidal *Nereocystis luetkeana* from 18 sites were collected from the WA State (US), and BC (Canada) coasts (Fig 1) of the Salish Sea; sixteen sites had both *F*. *distichus* and *N*. *luetkeana*. Sites represented a range of potential contamination. Samples were collected from sites known to be or suspected of being contaminated based on current or historic activities, including mines (e.g., Britannia Mine), smelters (e.g., Ruston Way in the Asarco’s Tacoma smelter plume), coal gasification plants and refineries (e.g., Rock Bay, Fidalgo Bay), ports, shipyards, and ferry terminals (e.g., Duke Point, Point Hope, Mukilteo Ferry, Tsawwassen Jetty, Smith Cove, Port Angeles, Victoria Jetty, Port of Everett), and present and former pulp and paper mills (e.g., Crofton Mills, Elk Falls, and Goodridge Mill). They also included sites at or near National Oceanic and Atmospheric Administration (NOAA) Mussel Watch or Washington Department of Fish and Wildlife (WFDW) transplanted (i.e., caged) mussel sites. These sites included Smith Cove, Mukilteo Ferry, Wing Point, Waterman Point, Four Mile Rock, Post Point, Port of Everett, and Ruston Way [35, 44, 45]. Additional sites included areas where seafood was historically or is currently gathered by Indigenous peoples on Canadian First Nation reserve lands, on Washington tribal lands, in “usual and accustomed” harvesting areas, and in “traditional territory” with minimal history of upland industrial development.

### 2.2 Sample collection and preparation

Sampling occurred from June to September 2015, during the growing season, with sustainable harvesting methods that followed the laws for recreational shellfish and seaweed license holders issued by WDFW and the 2015 regulations issued by the Aquatic Plants Program, Ministry of Agriculture, BC, Canada. All sites were sampled one time, except Vashon Island, which was sampled in June, July, and August. Beaches were divided into left, right, and center segments, and samples were collected from each segment. When relevant, field site access was approved by duly authorized First Nation and Indian Tribal Community staff. Permits were not obtained or required due to the specific nature of the work. Fidalgo Bay Aquatic Reserve seaweed site access was provided by Birdie Davenport. Botanical Beach Provincial Park seaweed site access was provided by Kristine Pearson, Pacheedaht First Nation. The Canadian Forces Base at Esquimalt Harbour provided access to seaweed sample sites in Esquimalt Harbour area. All other sites were publicly accessible or had permission as described above. Geographic or GPS (global positioning system) coordinates were recorded; however, we are not publishing the coordinates because of the sensitive nature of providing specific locations on Indian Reserve Lands or seaweed sites in First Nation or Tribe Territories.

*N*. *luetkeana* non-reproductive and reproductive blades were cut from individuals in the shallow subtidal zone, blades were removed at least 70 cm above the bulb with a ceramic-bladed knife. *F*. *distichus* and *F*. *spiralis* thalli, including blades, midribs, and reproductive and non-reproductive apical tips, were collected from the intertidal zone with a ceramic-bladed knife so that no more than half of any individual was removed. *F*. *spiralis* was only sampled when *F*. *distichus* was not present on a beach.

Seaweeds were rinsed with seawater at the sampling site in cotton mesh bags. The bags containing the seaweed were rinsed 12 times in seawater from the site, drained, and immediately transferred and stored in polyethylene (Ziploc^™^) bags in a cooler with ice packs. Within two days, they were transferred to a -80°C ultra-low freezer, except for three remote sampling locations where samples were on ice packs for up to four days. To make a single composite sample, seaweeds were thawed, and visible fauna and epiphytes were removed. Seaweeds were not rinsed before processing as is consistent with the practices of many consumers [46]. For each site, to create the composite sample, equal portions of samples from the left, right and middle segments of the beach/kelp bed were processed together by hand-chopping the mixture into a slurry with ceramic-blade knives. Between samples, equipment was cleaned with Tergazyme^®^ detergent and de-ionized water. Homogenized seaweed (approximately 20 g per composite sample per set of analyses) was transferred to certified-contaminant-free, amber glass jars with plastic lids, and stored at -80°C until it was shipped on ice 1–3 weeks later for chemical analyses.

### 2.3 Chemical analysis

A complete list of analytes and measured concentrations along with lower limits of quantitation (LOQs) are given in [47]. *F*. *spiralis and F*. *distichus* tissues (but not *N*. *luetkeana* due to financial constraints) were analyzed for 22 organochlorine pesticides, 40 PCBs, 15 PBDEs and 51 polycyclic aromatic hydrocarbons (PAHs) by the NOAA / Northwest Fisheries Science Center Environmental Chemistry Lab (Seattle, Washington, USA) using the gas chromatography/mass spectrometry (GC/MS) method and quality assurance plan of Sloan et al., [48, 49]. The mean concentrations of analytes in method blanks were below detection limits or at least ten times lower than the lowest field sample for which the measured concentration was above the detection limit [47]. *F*. *spiralis and F*. *distichus* tissues were also analyzed for the 15 PCDD/Fs with the WHO 2005 TEFs [50] and the dioxin and furan octa homologues by AXYS Analytical Labs (Sidney, BC, Canada; currently SGS AXYS) using USEPA Method 1613b in accordance with internal QA/QC policies and procedures. To measure concentrations of metals, *F*. *spiralis*, *F*. *distichus*, and *N*. *luetkeana* tissues were dried in a 95°C oven overnight and acid digested with trace metal grade HNO_3_ and HCl. Percent moisture was determined prior to the digestions. The digested samples were analyzed at the University of California (Davis, California, USA) for 17 metals (beryllium [Be], vanadium [V], chromium [Cr], manganese [Mn], iron [Fe], cobalt [Co], nickel [Ni], copper [Cu], zinc [Zn], As, selenium [Se], molybdenum [Mo], Cd, barium [Ba], Hg, thallium [Tl], Pb) using an Agilent 7700 ICP-MS in He collision mode. The LOD and LOQ were determined according to FDA Elemental Analysis guidelines for food and related products.

A variety of methods (e.g., [51]) have been proposed to handle values that fall below the lower limit of quantitation (LOQ) for the purpose of data analysis. These range from a highly conservative approach of setting these values to the LOQ to a much less conservative approach, from a human health perspective, of setting them to 0. Unless otherwise noted, we chose a moderately conservative approach and used ½ the LOQ for our analyses. Thus, if a sample was below the LOQ, one-half the LOQ was used for summed concentrations of the congeners we analyzed (e.g., Σ_40_PCBs, ΣPBDEs, ΣPCDD/F TEQs), calculations of means, standard deviations, and medians, and for statistical analyses, unless otherwise stated. TEQs were calculated with 2005 WHO TEFs from [50]. Methods for calculating total chlordanes, total DDTs, Σ_40_PCBs, total chrysenes, ΣPBDEs, and ΣPCDD/F TEQs are included in the S1 Text.

### 2.4 Screening level calculations

To assess potential health risks to consumers, we compared seaweed contaminant concentrations to calculated health-based screening levels for the 22 persistent organic pollutants (POPs), seven PAHs (Table 2), and eleven metals (Table 3) for which RfDs or oral CSFs exist. We calculated two screening levels based on a consumption rate of 5 g dry weight (DW) of seaweed per day, which is the typical amount consumed in a meal [52]. This rate is comparable to values reported for South Korea at 8.5 g/adult/day [53], India and China at 5.2 g/capita/day [54–56], and Japan at 4 to 10.4 g/adult/day [57, 58].

**Table 2:** Summary of screening levels and analytical results for organic contaminants.

	*Fucus distichus* (N = 38 sites)				*Fucus spiralis* (N = 3 sites)				Screening Levels	
Analyte	Percent >LOQ (Mean LOQ)	Mean (SD)	Median	Range	Percent >LOQ (Mean LOQ)	Mean (SD)	Median	Range	SL_RfD_	SL_CSF_
***Pesticides***										
α-HCH	3 (0.33)	0.18 (0.10)	0.17	<LOQ-0.68	0 (0.26)	0.12 (0.03)	0.10	<LOQ	---	2.5
β-HCH	0 (0.33)	0.16 (0.07)	0.17	<LOQ	0 (0.25)	0.12 (0.04)	0.11	<LOQ	---	8.9
γ-HCH	3 (0.33)	0.19 (0.17)	0.17	<LOQ-1.15	0 (0.25)	0.12 (0.04)	0.11	<LOQ	4800	---
Heptachlor	0 (0.33)	0.16 (0.07)	0.17	<LOQ	0 (0.25)	0.12 (0.04)	0.11	<LOQ	8000	3.6
HPE	0 (0.33)	0.16 (0.07)	0.17	<LOQ	0 (0.25)	0.12 (0.04)	0.11	<LOQ	208	1.8
HCB	0 (0.33)	0.16 (0.07)	0.17	<LOQ	0 (0.25)	0.12 (0.04)	0.11	<LOQ	12800	10
Total chlordanes	NA	1.31 (0.53)	1.33	<LOQ	NA	0.96 (0.29)	0.85	<LOQ	8000	46
Mirex	0 (0.33)	0.16 (0.07)	0.17	<LOQ	0 (0.25)	0.12 (0.04)	0.11	<LOQ	3200	---
Endosulfan	0 (0.62)	0.31 (0.16)	0.27	<LOQ	0 (0.90)	0.44 (0.13)	0.39	<LOQ	96000	---
Dieldrin	0 (0.33)	0.16 (0.07)	0.17	<LOQ	0 (0.25)	0.12 (0.04)	0.11	<LOQ	800	1
Aldrin	0 (0.33)	0.16 (0.07)	0.17	<LOQ	0 (0.25)	0.12 (0.04)	0.11	<LOQ	480	0.94
4,4’-DDD	3 (0.33)	0.23 (0.33)	0.17	<LOQ-2.13	33 (0.25)	0.17 (0.07)	0.16	<LOQ-0.25	---	67
4,4’-DDE	0 (0.34)	0.16 (0.07)	0.17	<LOQ	33 (0.25)	0.22 (0.15)	0.16	<LOQ-0.39	---	47
4,4’-DDT	3 (0.33)	0.17 (0.07)	0.17	<LOQ-0.36	0 (0.25)	0.12 (0.04)	0.11	<LOQ	8000	47
∑DDTs	NA	1.06 (0.54)	1.04	<LOQ-3.21	NA	0.87 (0.)	0.97	<LOQ-1.01	8000	47
***Sum of Polychlorinated biphenyls (***Σ_**40**_***PCBs)***										
<LOQ = 0	NA	5.64 (3.1)	4.66	2.64–19.6*	100	6.94 (5.0)	4.32	3.83–12.7*	320	8*
<LOQ = ½ LOQ	NA	10.8 (4.0)	7.74	5.77–21.2*	100	10.2 (3.6)	9.04	7.31–14.2*	320	8*
<LOQ = LOQ	NA	15.9 (6.0)	13.2	7.81–29.7*	100	13.4 (2.8)	14.2	10.3–15.7*	320	8*
***Polychlorinated dibenzodioxins/furans (PCDD/Fs) and polybrominated diphenyl ethers (PBDEs)***										
2,3,7,8-TCDD	5 (0.00017)	0.00023 (0.00013)	0.00018	<LOQ-0.00061	0 (0.00012)	0.00012 (0.00001)	0.00013	<LOQ	0.011	---
ΣPCDD/F TEQ	NA	0.00051 (0.00038)	0.00043	<LOQ-0.0027	NA	0.0011 (0.0013)	0.00042	0.00038–0.00258	0.011	---
PBDE 47	3 (0.33)	0.18 (0.11)	0.18	<LOQ-0.75	0 (0.25)	0.12 (0.04)	0.10	<LOQ	1600	---
PBDE 99	3 (0.33)	0.18 (0.11)	0.17	<LOQ-0.68	0 (0.25)	0.12 (0.04)	0.11	<LOQ	1600	---
PBDE 153	0 (0.33)	0.18 (0.11)	0.17	<LOQ	0 (0.25)	0.12 (0.04)	0.11	<LOQ	3200	---
ΣPBDEs		2.62 (1.03)	2.60	<LOQ-5.29		2.01 (0.33)	1.90	<LOQ-2.38	1600	---
***Polycyclic aromatic hydrocarbons (PAHs)***										
Acenaphthene	5 (1.83)	1.16 (1.28)	0.87	<LOQ-8.62	0 (1.87)	0.94 (0.08)	0.94	<LOQ	960000	---
Anthracene	5 (1.34)	1.60 (5.31)	0.64	<LOQ-34.1	33 (1.30)	1.65 (1.75)	0.69	<LOQ-3.67	4800000	---
Benzo[*a*]-pyrene	5 (1.60)	2.73 (10.3)	0.76	<LOQ-64.6*	66 (1.50)	9.40 (10.3)	6.62	<LOQ-20.8*	480	16*
Total chrysenes	NA	8.39 (24.1)	3.40	<LOQ-151	NA	38.0 (31.9)	44.7	<LOQ-66.0	---	220
Fluoranthene	13 (1.80)	8.09 (36.8)	0.86	<LOQ-82.6	66 (1.60)	26.2 (22.1)	37.2	<LOQ-40.7	640000	---
Fluorene	5 (1.80)	1.36 (2.53)	0.84	<LOQ-16.5	33 (1.70)	1.29 (0.77)	0.89	<LOQ-2.18	640000	---
Naphthalene	100 (---)	6.20 (7.90)	4.72	2.63–53.9	100 (---)	6.21 (3.66)	4.5	3.72–10.4	320000	---

Select analytes that have screening levels: percent of samples above lower limit of quantification (LOQ), mean of the LOQs for measurements below the LOQ, means, standard deviations (SD), medians, and ranges (in μg kg^-1^ DW), with screening levels (SLs; in μg kg^-1^ DW) based on reference dose (RfD) or cancer slope factors (CSF) from the USEPA Integrated Risk Information System database. Except for Σ_40_PCBs, values less than the LOQ were estimated as the LOQ/2 for mean, SD and median calculations. For Σ_40_PCBs, separate calculations were made estimating values less than the LOQ as either zero, ½ the LOQ, or the LOQ for each congener below its respective LOQ. HCH: hexachlorocyclohexane; HPE: heptachlor epoxide; HCB: hexachlorobenzene; NA: not applicable; TCDD: tetrachloro*-p-*dibenzo dioxin; TEQ: toxic equivalency (WHO, 2005).

*Analytes for which one or more samples were above an SL.

**Table 3:** Summary of screening levels and analytical results for metals.

	*Fucus distichus* (N = 38 sites)				*Fucus spiralis* (N = 3 sites)				*Nereocystis luetkeana* (N = 17 sites)				
Analyte	Percent >LOQ	Mean (SD)	Median	Range	Percent >LOQ	Mean (SD)	Median	Range	Percent >LOQ	Mean (SD)	Median	Range	SL_RFD_
Ba	100	12.3 (12.6)	9.96	5.16–87.6	100	21.0 (1.04)	20.8	20.2–22.2	100	11.6 (2.38)	11.5	7.06–15.6	3200
Be	0	0.05 (0.00)	0.05	<LOQ	0	0.05 (0)	0.05	<LOQ	0	0.05 (0.00)	0.05	<LOQ	32
Cd	100	2.52 (0.85)	2.47	1.15–4.25	100	2.03 (0.31)	2.02	1.73–2.34	100	5.69 (1.50)	5.51	2.48–7.91	16
Co	100	0.78 (0.69)	0.53	0.11–3.83	100	2.48 (1.99)	2.46	0.51–4.48	17	0.07 (0.04)	0.05	<LOQ-0.18	---
Cr	98	0.48 (0.88)	0.27	<LOQ-5.12	100	2.29 (2.81)	1.17	0.22–5.49	94	0.25 (0.10)	0.26	<LOQ-0.43	48 ^¥^
Cu	8	4.36 (20.9)	0.05	<LOQ-130	33	2.94 (5.00)	0.05	<LOQ-8.71	0	0.05 (0.00)	0.05	<LOQ	---
Fe	100	212 (476)	71.9	21.6–2485	100	1460 (1718)	884	104–3392	100	40.1 (15.6)	34.2	21.6–77.3	---
Hg	18	0.06 (0.04)	0.05	<LOQ-0.21	0	0.05 (0.00)	0.05	<LOQ	78	0.12 (0.05)	0.12	<LOQ-0.25	1.6^¥^
Mn	100	48.5 (40.9)	37.6	11.8–202	100	279 (276)	187	61.2–590	100	5.96 (0.77)	5.96	4.53–7.24	2240
Mo	95	0.21 (0.10)	0.19	<LOQ-0.64	100	0.26 (0.06)	0.24	0.22–0.33	100	0.24 (0.06)	0.24	0.16–0.40	80
Ni	65	1.60 (2.38)	0.70	<LOQ-11.6	100	5.34 (1.53)	4.74	4.21–7.08	0	0.05 (0.00)	0.05	<LOQ	320
Pb	25	0.66 (2.54)	0.05	<LOQ-13.2	66	4.62 (7.61)	0.41	<LOQ-13.4	11	0.06 (0.03)	0.05	<LOQ-0.14	---
Se	10	0.06 (0.02)	0.05	<LOQ-0.14	67	0.12 (0.06)	0.15	<LOQ-0.16	6	0.05 (0.01)	0.05	<LOQ-0.11	80
tAs	100	26.8 (4.96)	26.1	16.4–37.0	101	19.1 (0.54)	18.8	18.7–19.7	100	72.5 (12.0)	71.1	56.7–98.9	4.8^¥^^,^ *
Tl	0	0.05 (0.00)	0.05	<LOQ	0	0.05 (0.00)	0.05	<LOQ	0	0.05 (0.00)	0.05	<LOQ	---
V	100	0.94 (0.92)	0.70	0.46–5.12	100	3.10 (2.97)	2.01	0.83–6.46	100	1.27 (0.47)	1.22	0.76–2.72	144^¥^
Zn	100	39.4 (52.1)	20.1	10.6–229	100	98.1 (72.2)	68.0	45.8–181	100	24.0 (13.5)	18.1	11.4–63.1	4800

All metals: percent of samples above limits of quantitation (LOQs), means, standard deviations (SD), medians, and ranges (in mg^.^kg^-1^ DW) with screening levels (SLs) based on reference dose (RfD) from the USEPA IRIS database for concentrations (in mg^.^kg^-1^ DW). Values less than the LOQ were estimated as the LOQ/2. For all measurements below the LOQ, the LOQ was 0.1 mg^.^kg^-1^ DW [47].

* A SL_CSF_ for inorganic As was also calculated as 0.0107 mg/kg.

^¥^ The calculated screening level is based on an RfD for Cr (IV), methyl Hg, inorganic As, or V pentoxide.

The RfD-based screening level (SL_RfD_) for adults was based on RfDs (mg/kg body weight/day) obtained from the Integrated Risk Information System (IRIS) database provided by the USEPA and was calculated as:

$${\text{SL}}_{\text{RfD}}=\left(\left(\text{RfD}\;\text{x}\;\text{BW}\right)/\text{CR}\right)*1,000$$ (1)

where SL_RfD_ (in mg chemical/kg DW of seaweed) is the reference dose-based screening level and is used for non-carcinogenic effects, BW is body weight (assumed to be 80 kg), CR is the consumption rate (g DW of seaweed/day), and 1,000 is a unit correction. We did not speciate metals in our chemical analysis, however, in the IRIS database there are oral RfDs for five metal species (Cr (III), Cr (IV), methyl Hg, inorganic As, and V pentoxide). To be conservative, we used the RfD for the speciated forms of these metals to calculate the screening levels; for Cr, we used Cr (IV). The second screening level (Eq 2) was based on oral CSFs (1/mg/kg-day) for adults obtained from the USEPA IRIS database.

$${\text{SL}}_{\text{CSF}}=\left(\text{ARL}\;\text{x}\;\text{BW}\right)/\left(\text{CSF}\;\text{x}\;\text{CR}\right)$$ (2)

where SL_CSF_ (in mg chemical/kg DW seaweed) is the CSF-based screening level and is used for carcinogenic effects, and ARL is the acceptable risk level of 1E-6. The upper-bound slope factor in the higher risk and persistence tier was used for PCBs. For convenience, when making comparisons to organic contaminants, screening levels were converted to μg chemical/kg seaweed.

We then compared the seaweed contaminant concentrations to the calculated screening levels. To guide acceptable site-specific consumption rates for sites where contaminant concentrations exceeded screening levels, a new maximum consumption rate was calculated by solving for the consumption rate in Eqs 1 and 2 with the screening level set at the concentration (in mg/kg DW) that we measured in the seaweed. If more than one analyte exceeded the screening level at a site, the most conservative new maximum consumption rate was identified. In the case of Pb, which does not have an assigned RfD or CSF, we compared our seaweed concentrations to the lowest international limit (China: 1 mg/kg DW) and calculated new consumption rates with that value.

### 2.5 Total diet study

To provide context for the measured levels of focal contaminants in *F*. *distichus* and *N*. *luetkeana*, we calculated the amounts of contaminants that would be consumed in 5 g DW portions of seaweed and in established portion sizes for commonly consumed foods based on contaminant concentrations and portion sizes in [59–62]. We did not compare *F*. *spiralis* to commonly consumed foods due to limited samples (n = 3). To identify locations where a contaminant in seaweed is elevated compared to the mean, we flagged any samples where concentrations/portion exceeded twice the standard deviation for that species. These samples were considered statistical outliers and with only a few exceptions (e.g., Deep Bay, Vashon Island in June, and Jefferson Beach), they were collected near highly urbanized areas. The use of these statistical outliers was only for the total diet study comparisons. For each seaweed species and focal contaminant, we compared the concentration per portion in commonly consumed foods with the concentrations per portion of seaweed for samples contaminated enough to be identified as statistical outliers and with the averaged concentrations per portion of seaweed for all other remaining sites.

### 2.6 Interspecific comparisons

Comparisons of select metal concentrations in *F*. *distichus* and *N*. *luetkeana* were made with paired t-tests after testing for normality. The Bonferroni-corrected α value of 0.017 was used to account for making multiple comparisons.

### 2.7 Inclusivity in global research

Additional information regarding the ethical, cultural, and scientific considerations specific to inclusivity in global research is included in the S1 Checklist.

## 3.0 Results

### 3.1 Detection of organic compounds

**Fig 2:**
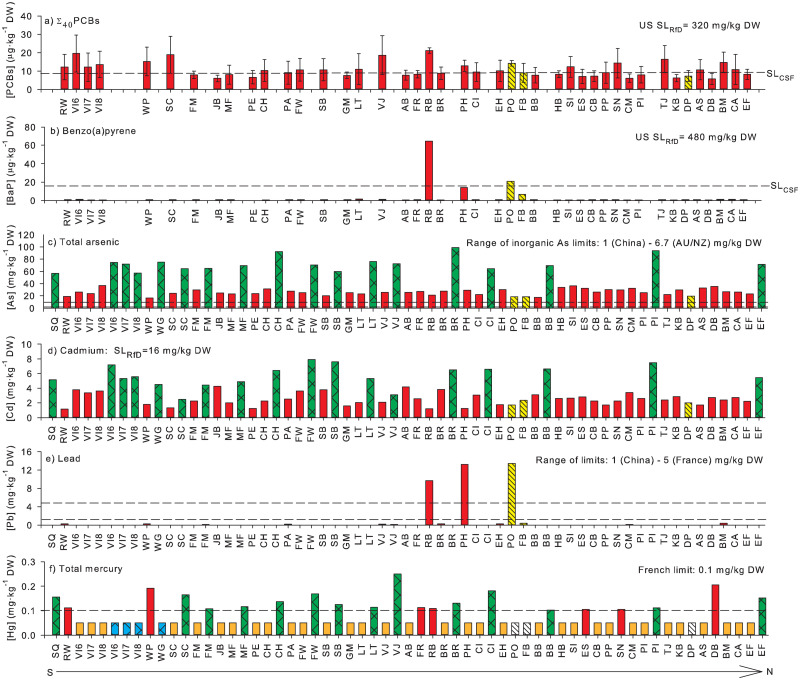
Concentrations of focal contaminants in *Fucus* spp. and *Nereocystis* at Salish Sea sites from the southernmost sites (left) to the northernmost sites (right). Bars are concentrations of a) Σ_40_PCBs (μg^.^kg^-1^ DW), b) benzo[*a*]-pyrene (BaP, μg^.^kg^-1^DW), c) total arsenic (mg^.^kg^-1^ DW), d) cadmium (mg^.^kg^-1^ DW), e) lead (mg^.^kg^-1^ DW), and f) total mercury (mg^.^kg^-1^ DW) calculated using the limit of quantification (LOQ)/2 when measured values were less than the LOQ. Upper ends of the vertical lines in a) are the calculated Σ_40_PCB concentrations when the LOQ was used for concentrations that were less than the LOQ. Lower ends of the bars are calculated values obtained when zero was used for the values less the LOQ. For a) Σ_40_PCBs and b) benzo[*a*]-pyrene, black dashed horizontal lines indicate the cancer slope factor-based screening levels (SL_CSF_). For c) total As, d) Cd, e) Pb, and f) total mercury, the upper black dashed horizontal line is the highest international limit (see Table 1) for the contaminant, and the lower black dashed horizontal line, if present, indicates the lowest international limit. Red and orange bars are concentrations in *Fucus distichus* that exceed or are less than the LOQ, respectively; yellow hatched bars or white hatched bars are concentrations in *Fucus spiralis* that exceed or are less than the LOQ, respectively; green cross-hatched or blue cross-hatched bars are concentrations in *Nereocystis luetkeana* that exceed or are less than the LOQ, respectively. Site codes are as in Fig 1.

Although some contaminants were at high enough concentrations to result in recalculated consumption rates, concentrations of organic contaminants in *Fucus* were generally low, with most samples below the analytes’ LOQs (Table 2 and [47]). For analyses of POPs and PAHs, LOQs for compounds that were below the LOQs ranged from 0.139 to 3.12 μg/kg; for PCDD/DFs, they ranged from 0.089 to 0.508 μg/kg. Fifteen percent of the pesticide measurements were above the LOQ (3311 total measurements; [47]). Of the pesticides that had screening levels (Table 2), concentrations of only four (α-HCH, γ-HCH, 4,4’-DDE, and 4,4’ DDT) were above LOQs in *F*. *distichus*, and this only occurred at Rock Bay. For *F*. *spiralis*, they were only above LOQs for 4,4’-DDD, and 4,4’-DDE at Portage Inlet. Only 26 percent of individual PCB congener measurements at all sites exceeded LOQs (1720 total measurements; [47]). In *F*. *distichus*, concentrations of 12 of the 40 PCB congeners were below LOQs at all sites, whereas in *F*. *spiralis*, 16 were below LOQs at all sites (Fig 2a). Only 35% of the PCDD/DF congener measurements at all sites exceeded LOQs (2884 total measurements; [47]). Most measurements of 2,3,7,8-TCDD, the only dioxin or furan congener with a screening level, were below LOQs, with only *F*. *distichus* at Ambleside and Deep Bay exceeding them (Table 2). Four of the PBDEs congeners were not able to be identified. Of the 11 identified congeners (PBDEs 28, 47, 49, 66, 85, 99, 100, 153, 154, 155, and 183), only two with screening levels (PBDE 47 and 99) exceeded their LOQs at one or more sites. PBDE 47 and 99 were above the LOQ at Rock Bay in *F*. *distichus* and PBDE 49 was above LOQs in 43% of the *F*. *distichus* samples and 66% of the *F*. *spiralis* samples. Similarly, concentrations of the PAHs with screening levels were below LOQs at most sites, except naphthalene, for which all samples were above LOQs (Table 2). Acenaphthene, anthracene, and fluorene concentrations were above LOQs in *F*. *distichus* at two sites in Victoria Harbour (Point Hope and Rock Bay), and anthracene and fluorene concentrations were above LOQs in *F*. *spiralis* at Portage Inlet, which is also in Victoria Harbour. Fluoranthene concentrations were above LOQs at the three inner Victoria Harbour sites (*F*. *spiralis* at Portage Inlet, and *F*. *distichus* at Rock Bay and Point Hope), and also in *F*. *spiralis* at Fidalgo Bay, and in *F*. *distichus* at Crofton Mill and Port Angeles. BaP concentrations exceeded LOQs in *Fucus* spp. at the three Victoria Harbour sites and in *F*. *spiralis* at Fidalgo Bay (Fig 2b).

### 3.2 Screening levels for organic chemicals

Except for Σ_40_PCBs and BaP, concentrations of all organic contaminants were below the RfD and CSF-based screening levels at all sites (Table 2). BaP concentrations only exceeded the CSF-based screening level of 16 ng/g^-1^ dry weight (Fig 2b) at two of the 41 sites: Portage Inlet and Rock Bay. None of the Σ_40_PCBs concentrations exceeded the RfD-based screening level regardless of how congener concentrations below the LOQ were treated. The number of sites with concentrations of Σ_40_PCBs that exceeded the CSF-based screening level, however, depended on how concentrations below the LOQ were treated (Table 2). When Σ_40_PCBs were calculated using 0 for congener concentrations less than the LOQ, 18% of *F*. *distichus* and 33% of *F*. *spiralis* samples had concentrations that exceeded the CSF-based screening level (Fig 2a). When they were calculated using ½ the LOQ for values less than the LOQ, 70% of *F*. *distichus* and 67% of *F*. *spiralis* samples had concentrations that exceeded the CSF-based screening level. Finally, when Σ_40_PCBs were calculated with values less than the LOQ set to the LOQ, 98% of *F*. *distichus* and all *F*. *spiralis* concentrations exceeded the CSF-based screening level. The timing of sampling could also affect whether screening levels were exceeded as Σ_40_PCBs concentrations in *F*. *distichus* calculated with values below the LOQ set to zero at Vashon Island were above the CSF-based screening level in June (VI6 in Fig 2a), but below it in July (VI7) and August (VI8); however, further work is needed to confirm a temporal pattern because these measurements were not replicated and were only made at one site.

Other organic contaminants in the seaweed samples were well below screening levels. For example, organochlorine pesticide concentrations at Rock Bay and Portage Inlet, which were among the highest concentrations we measured, were only 0.4–27% of the CSF-based screening levels or 0.005–0.04% of the RfD-based screening levels (Table 2). Only two samples had detectable 2,3,7,8-TCDD concentrations, but those concentrations were less than 0.1% of the screening level. Likewise, the ΣPCDD/F TEQs were well below the RfD-based screening level at all sites. Concentrations of ΣPBDEs and all PBDE congeners for which RfDs exist (PBDE 47, 99, and 153) were always less than 0.1% of those screening levels for *F*. *distichus* and *F*. *spiralis* at all sites (Table 1). Furthermore, maximum concentrations of acenaphthene, anthracene, fluorene, and naphthalene also did not exceed 0.002% of the RfD-based screening levels. The highest concentration of total chrysenes was 69% of the CSF-based screening level, and the highest fluoranthene concentration was 0.013% of the RfD-based screening level (Table 1).

### 3.3 Detection of metals

Concentrations of many metals (Ba, Cd, Co, Cr, Fe, Mn, Mo, total As, V and Zn) were above LOQs at most sites. However, Cu, Pb, and Se concentrations were generally below LOQs. For all metal analyses with a measured value below the LOQ, the LOQ was 0.1 mg/L [47]. Concentrations of Be and Tl in all three species were always below LOQs. Ni concentrations were above the LOQ in 65% and 100% of the *F*. *distichus* and *F*. *spiralis*, respectively, and none of the *N*. *luetkeana*. Conversely, total Hg was above the LOQ in 78% of the *N*. *luetkeana* compared to 18 and 0% of the *F*. *distichus* and *F*. *spiralis*, respectively (Table 3).

### 3.4 Screening levels and international limits for focal metals

An RfD or CSF for Pb has not been assigned, so comparisons to the calculated screening levels were not conducted. Except for As, the remaining metal concentrations never exceeded US RfD-based screening levels at any site (Table 3). Total As concentrations in all samples exceeded the US RfD- and CSF-based screening levels for inorganic As, as well as the recommended levels for inorganic As in China, France, and Australia/New Zealand (Fig 2c; Table 1). All Cd concentrations were above the levels recommended by Australia/New Zealand, France, and China (Fig 2d, Table 1). However, concentrations of Cd in all three species never exceeded the European Commission maximum level of 3 mg^.^kg^-1^ wet weight, assuming an 85% moisture content for the *N*. *luetkeana* and using actual wet:dry weight ratios for *Fucus* spp. Chinese and French recommended levels for Pb were exceeded only in Point Hope and Rock Bay for *F*. *distichus* and Portage Inlet for *F*. *spiralis* but were never exceeded in *N*. *luetkeana* samples (Fig 2e, Table 1). Total Hg did not exceed the screening level calculated with the RfD for methyl Hg in any samples (Fig 2f, Table 3), however, detectable total Hg concentrations exceeded the French recommended level of 0.1 mg^.^kg^-1^ DW for Hg in 15% of *F*. *distichus* samples and 78% of *N*. *luetkeana* samples. They did not exceed the French recommended level for Hg in any *F*. *spiralis* samples (Fig 2f, Table 1).

### 3.5 Interspecific, temporal, and latitudinal differences in metal concentrations

**Fig 3:**
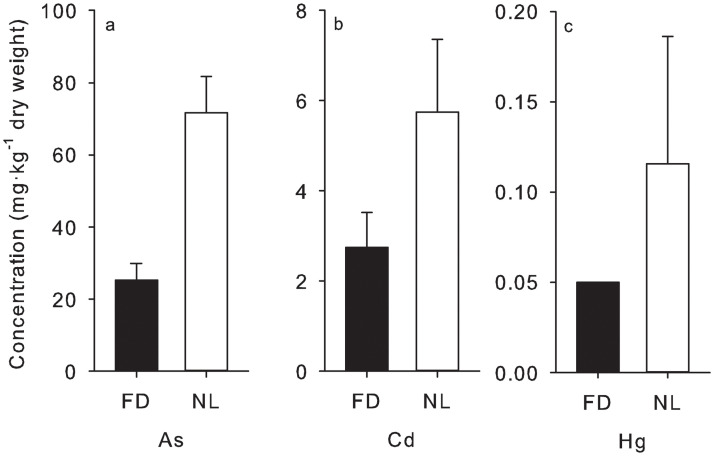
Concentrations (in mg^.^kg^-1^ DW) of a) total arsenic (As), b) cadmium (Cd), and c) total mercury (Hg) in *Fucus distichus* (FD) and *Nereocystis luetkeana* (NL) at sites where both species were collected (N = 16). Data are means ± 1 SD. Concentrations are significantly (P<0.001) higher in *N*. *luetkeana* than *F*. *distichus* for all three metals (paired t-tests).

Concentrations of total As, Cd, and total Hg were 184, 111, and 151% higher, respectively, in *N*. *luetkeana* than in *F*. *distichus* (Fig 3) when they were sampled from the same sites at the same time (N = 15 sites, paired t-tests: total As t = 16.25, P<0.001; Cd t = 10.13, P<0.001; total Hg t = 6.34, P<0.001). We saw evidence of temporal variability in total As and Cd concentrations in both *F*. *distichus* and *N*. *luetkeana* at Vashon Island (Fig 3), which were sampled in June, July and August; however, because these were single composite samples, it is not possible to determine whether these differences were significant. Although concentrations of many contaminants differed among sites, there were no obvious latitudinal patterns in their abundances (Fig 2). Correlations between latitude and total As, Cd, total Hg, Pb, BaP and Σ_40_PCBs in *F*. *distichus* were never statistically significant (|Spearman’s rho|<0.293, P>0.130). No statistically significant correlations existed between latitude and total As, Cd, total Hg, and Pb in *N*. *luetkeana* (|Spearman’s rho|<0.281, P>0.431). Likewise, collection dates were not significantly correlated with total As, Cd, total Hg, Pb, BaP and Σ_40_PCBs in *F*. *distichus* (|Spearman’s rho|<0.188, P>0.339), nor with total As, Cd, total Hg, and Pb in *N*. *luetkeana* (|Spearman’s rho|<0.804, P>0.090).

## 4.0 Discussion

Many of the contaminants measured in our collected seaweeds were below detection limits. At the majority of our sites, most concentrations of contaminants that have RfDs or CSFs were less than our calculated human health-based screening levels. However, our samples did have Σ_40_PCBs and BaP concentrations that exceeded the calculated screening levels, which resulted in calculating new consumption recommendations (Table 4). Concentrations of metals also commonly exceeded at least one of the limits recommended by countries outside of North America for Cd, Hg, and Pb. Arsenic concentrations are high in some Salish Sea finfish and shellfish [63], and therefore are of interest in the region. For these reasons, we limit our discussion of the distributions and impacts of these seaweed contaminants to the six focal contaminants: PCBs, BaP, Cd, Pb, Hg, and As.

**Table 4:** Recommended consumption rates (g DW/day) based on concentrations of Cd, Hg, Pb, benzo[*a*]pyrene (BaP) and Σ_40_PCBs measured at each site in this study.

Collection Site	Species	Collection Date	Cd (16)	Hg (1.6)	Pb (5)	BaP (16)	Σ_40_ PCBs (8)
Agate Beach, Lopez Island, WA (AB)	*F*. *distichus*	08/17/15	≥5	≥5	≥5	≥5	≥5
Ambleside, Vancouver, BC (AS)	*F*. *distichus*	09/03/15	≥5	≥5	≥5	≥5	**3.7**
Botanical Beach, Port Renfrew, BC (BB)	*F*. *distichus*	07/04/15	≥5	≥5	≥5	≥5	≥5
	*N*. *luetkeana*	07/04/15	≥5	≥5	≥5	---	---
Britannia Mine, Brittania Beach, BC (BM)	*F*. *distichus*	08/31/15	≥5	≥5	≥5	≥5	**2.7**
Brothers Islands, /Duntze Head, Victoria, BC (BR)	*F*. *distichus*	08/07/15	≥5	≥5	≥5	≥5	**4.5**
	*N*. *luetkeana*	08/07/15	≥5	≥5	≥5	---	---
Camano Head, Camano Island, WA (CH)	*F*. *distichus*	07/07/15	≥5	≥5	≥5	≥5	**3.9**
	*N*. *luetkeana*	07/07/15	≥5	≥5	≥5	---	---
Cape Mudge, Quadra Island, BC (CA)	*F*. *distichus*	09/02/15	≥5	≥5	≥5	≥5	**3.7**
Chatham Islands/*Tl’chés*, BC (CI)	*F*. *distichus*	06/05/15	≥5	≥5	≥5	≥5	**4.2**
	*N*. *luetkeana*	06/05/15	≥5	≥5	≥5	---	---
Coles Bay, North Saanich, BC (CB)	*F*. *distichus*	06/04/15	≥5	≥5	≥5	≥5	≥5
Crofton Mill, Crofton, BC (CM)	*F*. *distichus*	08/05/15	≥5	≥5	≥5	≥5	≥5
Deep Bay, Vancouver Island, BC (DB)	*F*. *distichus*	08/13/15	≥5	≥5	≥5	≥5	≥5
Duke Point Indust. Park, Nanaimo, BC (DP)	*F*. *spiralis*	09/02/15	≥5	≥5	≥5	≥5	≥5
East Sound, Orcas Island, WA (ES)	*F*. *distichus*	08/16/15	≥5	≥5	≥5	≥5	≥5
Elk Falls Pulp Mill, Campbell River, BC (EF)	*F*. *distichus*	09/01/15	≥5	≥5	≥5	≥5	**4.9**
	*N*. *luetkeana*	09/01/15	≥5	≥5	≥5	---	---
Esquimalt Harbour, Victoria, BC (EH)	*F*. *distichus*	08/07/15	≥5	≥5	≥5	≥5	**4.0**
Fidalgo Bay, Anacortes, WA (FB)	*F*. *spiralis*	08/26/15	≥5	≥5	≥5	≥5	**4.4**
Fort Rodd Hill, Victoria, BC (FR)	*F*. *distichus*	08/02/15	≥5	≥5	≥5	≥5	**4.8**
Four-Mile Rock, Seattle, WA (FM)	*F*. *distichus*	07/01/15	≥5	≥5	≥5	≥5	≥5
	*N*. *luetkeana*	07/01/15	≥5	≥5	≥5	---	---
Freshwater Bay, Clallum County, WA (FW)	*F*. *distichus*	07/03/15	≥5	≥5	≥5	≥5	**3.7**
	*N*. *luetkeana*	07/03/15	≥5	≥5	≥5	---	---
Goodridge Mill, Sooke, BC (GM)	*F*. *distichus*	08/10/15	≥5	≥5	≥5	≥5	≥5
Hagan Bight, Saanichton, BC (HB)	*F*. *distichus*	07/30/15	≥5	≥5	≥5	≥5	**4.8**
Jefferson Beach, Kingston, WA (JB)	*F*. *distichus*	06/30/15	≥5	≥5	≥5	≥5	≥5
Kulleet Bay, Vancouver Island, BC (KB)	*F*. *distichus*	08/12/15	≥5	≥5	≥5	≥5	≥5
Lone Tree Point, Fidalgo Island, WA (LT)	*F*. *distichus*	08/25/15	≥5	≥5	≥5	≥5	**3.6**
	*N*. *luetkeana*	08/25/15	≥5	≥5	≥5	---	---
Mukilteo Ferry, Mukilteo, WA (MF)	*F*. *distichus*	06/16/15	≥5	≥5	≥5	≥5	**5.0**
	*N*. *luetkeana*	06/16/15	≥5	≥5	≥5	---	---
Penelakut Island, BC (PI)	*F*. *distichus*	07/05/15	≥5	≥5	≥5	≥5	≥5
	*N*. *luetkeana*	07/05/15	≥5	≥5	≥5	≥5	≥5
Point Hope Shipyard, Victoria, BC (PH)	*F*. *distichus*	07/18/15	≥5	≥5	**1.9**	≥5	3.1
Port Angeles, WA (PA)	*F*. *distichus*	07/17/15	≥5	≥5	≥5	≥5	**4.4**
Portage Inlet, Victoria, BC (PO)	*F*. *spiralis*	08/03/15	≥5	≥5	**1.9**	3.8	2.8
Pigeon Creek Park, Everett, WA (PE)	*F*. *distichus*	08/27/15	≥5	≥5	≥5	≥5	≥5
Post Point, Bellingham Bay, WA (PP)	*F*. *distichus*	07/29/15	≥5	≥5	≥5	≥5	**4.4**
Rock Bay, Victoria, BC (RB)	*F*. *distichus*	07/18/15	≥5	≥5	2.6	**1.2**	1.9
Ruston Way, Tacoma, WA (RW)	*F*. *distichus*	09/06/15	≥5	≥5	≥5	≥5	**3.3**
Sansum Narrows, North Cowichan, BC (SN)	*F*. *distichus*	08/04/15	≥5	≥5	≥5	≥5	**2.8**
Senanus Island, BC (SI)	*F*. *distichus*	07/30/15	≥5	≥5	≥5	≥5	**3.3**
Smith Cove, Seattle, WA (SC)	*F*. *distichus*	06/02/15	≥5	≥5	≥5	≥5	**2.1**
	*N*. *luetkeana*	06/02/15	≥5	≥5	≥5	---	---
Sooke Bay, Sooke, BC (SB)	*F*. *distichus*	08/09/15	≥5	≥5	≥5	≥5	**3.7**
	*N*. *luetkeana*	08/09/15	≥5	≥5	≥5	---	---
Squaxin Island, Shelton, WA (SQ)	*N*. *luetkeana*	06/18/15	≥5	≥5	≥5	---	---
Tsawwassen Jetty, Delta, BC (TJ)	*F*. *distichus*	08/10/15	≥5	≥5	≥5	≥5	**2.4**
Vashon Island, WA (VI)	*F*. *distichus*	06/02/15	≥5	≥5	≥5	≥5	**2.0**
	*F*. *distichus*	07/02/15	≥5	≥5	≥5	≥5	**3.3**
	*F*. *distichus*	08/14/15	≥5	≥5	≥5	≥5	**2.9**
	*N*. *luetkeana*	06/02/15	≥5	≥5	≥5	---	---
	*N*. *luetkeana*	07/02/15	≥5	≥5	≥5	---	---
	*N*. *luetkeana*	08/14/15	≥5	≥5	≥5	---	---
Victoria Jetty, Victoria, BC (VJ)	*F*. *distichus*	07/18/15	≥5	≥5	≥5	≥5	**2.1**
	*N*. *luetkeana*	07/18/15	≥5	≥5	≥5	---	---
Waterman Point, WA (WP)	*F*. *distichus*	06/30/15	≥5	≥5	≥5	≥5	**2.6**
Wing Point, Bainbridge Island, WA (WG)	*N*. *luetkeana*	06/30/15	≥5	≥5	≥5	---	---

Bolded values are the lowest consumption rate for a species at a particular site if the lowest value is less than 5 g dry weight/day. Values in parentheses below contaminant types are screening levels (in μg/kg for Σ_40_PCBs and BaP and in mg/kg for Cd, Hg, and Pb), based on USEPA CSFs (PCBs and BaP) or RfDs (Cd and methyl Hg). The French limit for Pb was used as USEPA RfDs for Pb do not exist. Σ40PCB values were based on calculations that used ½ the LOQ when values were less than the LOQ.

### 4.1 Contaminant concentrations

Many chemicals that can biomagnify were measured at low concentrations in our seaweed samples possibly because of the seaweeds’ annual (*Nereocystis*) or biennial (*Fucus* spp.) life cycles, low lipid content, and position at the bottom of the food web. However, PCBs were detected in all our *Fucus* and *Nereocystis* samples, indicating that they are ubiquitous in edible seaweeds throughout the Salish Sea. PCBs are of high concern in the Salish Sea because they occur in a wide range of species at concentrations high enough to cause concern for both the marine organisms and humans who consume them. When our Σ_40_PCB concentrations for *F*. *distichus* were compared to the Venice Lagoon (Italy), the only location for which comparable data for *Fucus* spp. could be found, those in the Salish Sea were higher (S1 Table).

Even if our Σ_40_PCBs are calculated using 0 for congeners below LOQs, all sites in the current study had higher concentrations than the average of 3 μg/kg DW reported for an unidentified species of *Fucus* [64] (likely *F*. *virsoides* based on distribution information from [65]). BaP, the other focal organic contaminant, was only detected in seaweeds collected at 5 sites in the Salish Sea, and generally fell within the ranges reported for *Fucus* spp. elsewhere, although the Salish Sea site with the highest BaP concentrations, Rock Bay, had a concentration (64.6 μg/kg DW) slightly higher than the maximum value found in *F*. *vesiculosus* (64 μg/kg) from Norway [66]. It should be noted that comparisons to literature-reported seaweed concentrations come with caveats. Sites from these other studies may have been selected to represent extreme levels of contamination, different analytical techniques may have been used, data analysis is not consistent, interspecific variation in uptake rates may occur, and there are limited studies for some of the contaminants we investigated [67]. A comparison is useful, however, as a way of generally assessing whether Salish Sea seaweeds demonstrate an extreme level of contamination. Details on how literature was selected for comparison are in the S2 Text.

In contrast to the organic chemicals, many metals were above LOQs at most of our sites. Higher concentrations of metals are predicted as seaweeds have been shown to hyper-accumulate metals [68–70]. Brown seaweeds are highly effective at bioabsorbing and bioaccumulating metals due to their outer cell walls and matrix structures, which have abundant mucilaginous polysaccharides such as alginate and fucoidans and metal-binding metallothionein proteins and phlorotannins [69, 71]. Like PCBs, the focal contaminants Cd and total As were detected in all our *Fucus* and *Nereocystis* samples, supporting that they too are widely distributed in these Salish Sea seaweeds. However, total Hg and Pb were not commonly detected, with only 18 and 25% of samples above the limits of quantification.

The average concentrations of Cd, Pb, and total As in *F*. *distichus* and *F*. *spiralis* (Table 2) were within ranges of these metals measured in *Fucus* spp. in other studies (S2 Table). Likewise, Pb and total As in *N*. *luetkeana* are comparable to those in previous studies (S2 Table). At the sites where Hg was detectable (Fig 2f), our concentrations of total Hg in *Fucus* were higher than previously reported (S1 Table), except for a single report for *F*. *vesiculosus* in Norway [72], which had total Hg concentrations that exceeded our highest measurement by more than fourfold. Average total Hg concentrations in *N*. *luetkeana* (0.12 mg/kg) were also higher than in earlier reports from British Columbia where Hg was below detection levels [72, 73]. Average Cd concentrations (5.69 mg/kg) in *N*. *luetkeana* (Table 3) were 2 to 19 times greater than previously published concentrations (S2 Table) from British Columbia [72, 73].

Some of these differences may be attributed to our study design, which included sites ranging from those with minimal history of upland industrial development to sites known to be contaminated. The differences may also be the result of the Salish Sea’s hydrology and geological history, which causes background concentrations of many elements, such as As and Cd, to be elevated [74, 75], and affects their concentrations in marine biota. For example, clams, crabs, and finfish in Puget Sound generally have high levels of inorganic As, likely from natural sources, such as groundwater runoff from areas with volcanic activity [76]. Likewise, high Cd concentrations in mussels from the Salish Sea and other sites off the coast of Washington have been attributed to the upwelling of Cd-rich waters [77]. However, natural sources are not the only concern as sediment core profiling at two sites in Puget Sound has shown that many elements, such as As, Pb and Cu, have increased as a result of urbanization of the surrounding watershed [78]. Naturally occurring metals, therefore, are expected to contribute to a baseline concentration of metals in the seaweed with elevated levels near urbanized and industrial areas.

**Fig 4:**
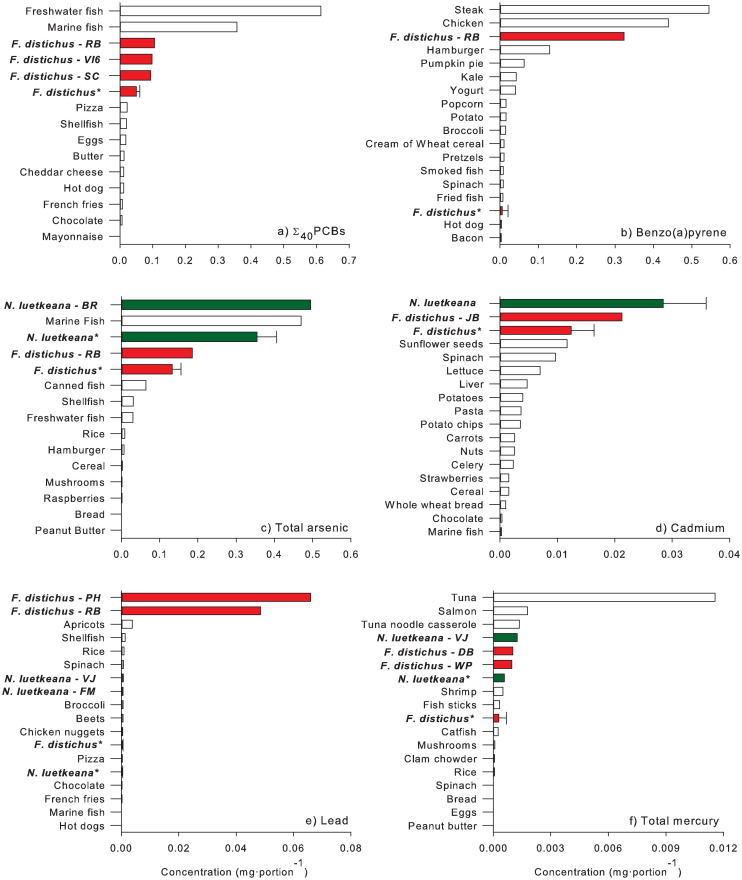
Comparisons of a) Σ_40_PCBs, b) benzo[*a*]-pyrene, c) total arsenic, d) cadmium, e) lead, and f) mercury in *F*. *distichus* (red bars) and *N*. *luetkeana* (green bars) to commonly consumed foods. Sites for which contaminant concentrations exceeded the mean + 2SD are shown separately. Bars labeled as *F*. *distichus* or *N*. *luetkeana* are mean concentrations (+ 1SD) from all sites that do not exceed the mean + 2SD. *Means that excluded sites with values greater than the mean + 2SD. Data for commonly consumed foods were obtained from [59–62]. More information about commonly consumed foods and the sources for this data are provided in S3 Table.

It should be noted that average concentrations of focal contaminants were sometimes skewed by a few sites that had high concentrations, which were identified as statistical outliers for our diet comparisons (Fig 4). These included Σ_40_PCB levels in *F*. *distichus* at Rock Bay, Vashon Island (June only), and Smith Cove; BaP in *F*. *distichus* at Rock Bay; total As in *F*. *distichus* at Vashon Island (August only); total As in *N*. *luetkeana* at Brothers Islands/Duntze Head; Cd in *F*. *distichus* at Jefferson Beach; Pb in *F*. *distichus* at Point Hope and Rock Bay; Pb in *N*. *luetkeana* at Four Mile Rock and Victoria Jetty; total Hg in *F*. *distichus* at Deep Bay and Waterman Point; and, total Hg in *N*. *luetkeana* at Victoria Jetty. All sites except Deep Bay (DB) on central Vancouver Island were in South Puget Sound near Seattle (US) or in Victoria/Esquilmalt Harbour (Canada). These areas have a history of heavy industrialization, are highly urbanized with high amounts of impervious surfaces, and are influenced by large drainage basins.

As a result of historic activities, the region includes several known or suspected contaminated sites. An example in the United States Salish Sea is the Lower Duwamish Waterway (LDW) in South Puget Sound which is downstream of the Green River Basin and flows into Elliott Bay. A five-mile stretch of this waterway is a Federal USEPA Comprehensive Environmental Response, Compensation, and Liability Act (CERCLA) Site with PCBs, carcinogenic PAHs, and Hg in sediments at concentrations that exceed acceptable levels for human health [79]. Seventeen formal Washington State cleanup Sites under the Model Toxics Control Act, the state environmental cleanup law, are part of the 20,400-acre LDW source control area [80]. In Canada, the lower portion of Victoria Harbour was a shipping and industrial port but has evolved to serve recreational boating and mooring, commercial and light industry, and residential needs as well. Portage Inlet, in the upper Harbour, was not heavily industrialized because of limited navigable waters, but is surrounded by residential areas and used by small pleasure craft. Industrial contributions to contaminants in Esquimalt Harbour have included sawmills, and the presence of Royal Navy and ship servicing activities [81, 82].

Although historic and current industrial activities are well-established sources of persistent environmental contaminants such as PCBs and metals, there is a growing recognition that nonpoint source pollution is a major and current contributor to marine contamination. For example, surface runoff, which includes stormwater, nonpoint source overland flow and groundwater discharge to surface waters, is the largest contributor of PCBs, PAHs, pesticides and metals to Puget Sound [83, 84]. Contaminant mass loading is also affected by drainage basin size and types of land use. Large areas of forested lands contribute high volumes of surface runoff but low concentrations of contaminants, whereas small urbanized and agricultural areas contribute smaller volumes but higher contaminant concentrations [85]. Likewise, modeling and empirical studies of Victoria (Canada) indicate that stormwater runoff (defined here to include surface runoff, precipitation, snowmelt and groundwater that is hydrologically connected to stormwater drainage systems) could be the primary route for surface water contaminants including metals and organic chemicals, such as PCBs, in Victoria Harbour [86, 87] and could contribute to high contaminant concentrations in seaweeds near Victoria.

### 4.2 Dietary implications

In this study, we calculated the amounts of *F*. *distichus* and *N*. *luetkeana* from individual sites that could be consumed without exceeding the following limits: CSF-based screening levels for Σ_40_PCBs, BaP, and RfD-based screening levels for Cd or methyl Hg from the risk-based USEPA recommendations; or international limits in seaweeds for Pb. We did not complete these calculations for As given the uncertainties in the amount of inorganic As in the samples. In our analysis, we compared five of the focal contaminant concentrations in seaweeds to RfD and CSF-based screening levels, where the CSF is specifically related to cancer risk. US regulations use 1 x 10^−4^ to 1 x 10^−6^ for an acceptable excess lifetime cancer risk, with the latter being the most protective. The choice of the risk level to use is a policy decision as opposed to a scientific decision. The CSFs we used are for an acceptable cancer risk at 1 x 10^−6^ but are based on a standard methodology that likely overestimates cancer risk by several orders of magnitude. This results in an estimate of risk that is more protective than the 1 x 10^−6^ cancer risk [88]. Additionally, there are different levels of confidence for whether a chemical is a human carcinogen. Contaminants are classified into categories based on the confidence that they cause cancer in humans proceeding from high to low evidence levels: “known human carcinogens”, “probable human carcinogens”, and “possible human carcinogens” [89]; these classifications were updated with new descriptors such as “carcinogenic to humans” to replace “known human carcinogens” [90]. Finally, there is concern that unintended consequences, such as avoiding a food with potential health benefits, may result from perceived concerns about potential cancer risk. Many of these considerations for fish consumption are appropriate for seaweed, which present consumers with a trade-off decision between potential health benefits and cultural well-being, and potential health risks from contaminant exposure.

Of all contaminants for which we could calculate screening levels based on USEPA information, only Σ_40_PCBs and BaP had samples with exceedances. This is in agreement with a number of studies that have identified PCBs and high molecular weight PAHs as contaminants of high concern in the Salish Sea, regarding both organismal and human health. PCBs have long been monitored in a number of Puget Sound indicator species of ecosystem health including English sole, a benthic flatfish (*Parophrys vetulus*), Pacific herring, a pelagic planktivore (*Clupea pallasii*–[36], bay mussels, a nearshore bivalve (*Mytilus trossulus*–[35, 45]), and Chinook salmon (*Oncorhynchus tshawytscha*) in both juvenile [91] and adult [92] forms. The Washington State Department of Health recommends meal limits on salmon and other Puget Sound species because of high PCB levels [93], and PCBs are a primary target for reductions in Puget Sound to restore ecosystem health [94].

PCBs, which are environmentally persistent and a “probable human carcinogen” as opposed to a “known carcinogen” [95], were above lower limits of quantitation in *Fucus* spp. at all our sites. While Σ_40_PCBs concentrations at many sites exceeded the CSF-based screening level, they were still lower in an established portion size of seaweed than concentrations in other commonly consumed foods (Fig 4a, S3 Table). Even at Rock Bay, the site with the highest Σ_40_PCBs, concentrations of PCBs per portion were only 17–29% of those reported in marine and freshwater fish, although they were higher than in other food types. One of the limitations to our approach is that we sum the 40 PCB congeners, where the CSF and RfD are based on total PCBs. To consider this limitation, we also used the West et al. [36] fish-based calculation for a subset of our congeners to estimate total PCBs (S1 Fig). The comparison to screening levels resulted in an additional four sites exceeding the RfD-based screening level and requiring a lowered recommended consumption rate (S4 Table). These sites and others would have exceeded the screening level had we used the LOQ instead of half the LOQ when concentrations were below the LOQ, demonstrating the uncertainty associated with the considerations of PCBs.

High molecular weight PAHs such as BaP or diseases caused by BaP exposure have also been monitored in English sole [94, 96] and two crustacean species: the Dungeness crab *Metacarcinus magister* and the spot prawn *Pandalus platyceros* [97]. These chemicals also have been reported in intertidal seagrasses (*Zostera marina*) near a shoreline oil refinery [98] and in developing embryonic Pacific herring transplanted near creosote-treated pilings in the nearshore, at levels high enough to impair their health [99].

Concentrations of BaP, classified as “carcinogenic to humans” [100], did not exceed the RfD-based screening level and only exceeded the USEPA CSF-based screening level (16 μg/kg) in two samples from Victoria Harbour: *F*. *distichus* at Rock Bay and *F*. *spiralis* at Point Hope (Fig 2b). When calculated as a concentration per portion, the high BaP concentrations in Rock Bay *F*. *distichus* made this sample a significant statistical outlier (Fig 4). As a result, BaP concentrations in *F*. *distichus* from Rock Bay were comparable to grilled, very well-done steaks and grilled, well-done chicken with bone and skin, foods with some of the highest reported BaP concentrations in commonly consumed foods (Fig 4b, S3 Table). Otherwise, average BaP concentrations/portion in *F*. *distichus* were lower than those in many commonly consumed foods (Fig 4b).

Concentrations of total As also frequently exceeded screening levels for inorganic arsenic, a “known human carcinogen” [95]. Furthermore, our diet analysis found that average total As concentrations in *F*. *distichus* and *N*. *luetkeana* exceeded concentrations per portion in commonly consumed foods except for marine fish (Fig 4c). Without knowing the relative proportions of organic and inorganic As species in our samples, the exceedances of the screening levels and their implications for consuming Salish Sea seaweeds are difficult to interpret. Studies that speciated As in seaweeds from BC found the inorganic As content to be 38% of total As in *Fucus* sp. [101] and 1–3% in *N*. *luetkean*a [102]. However, inorganic As in the genus *Sargassum*, including “Hijiki” which is sold commercially, are high enough that consumption advisories were issued regarding its use by the United Kingdom, New Zealand, Australia, China and Canada [103–105]. *Sargassum muticum*, an invasive, commonly harvested Salish Sea seaweed is also relatively high in inorganic arsenic [102]. Seaweed, fish, and shellfish are known to be high in arsenosugars [106]. Until recently, the toxicity of arsenosugars was thought to be of little concern [56, 101]. However, arsenosugars may be metabolized into potentially toxic metabolites, such as thio-dimethyl arsenic, that can have cytotoxic effects on the bladder and lungs [107, 108].

High levels of Cd can cause bone fragility, reduced lung function, and kidney damage [109]. Cd concentrations in Salish Sea *F*. *distichus* and *N*. *luetkeana* were uniformly below US RfD-based screening levels (Table 3), but consistently above international limits for seaweed (Table 1). Average Cd concentrations per portion in *F*. *distichus* and *N*. *luetkeana* were higher than in other commonly consumed foods (Fig 4d; S3 Table) but were lower than in shellfish from the Salish Sea. Concentrations of Cd in Salish Sea shellfish from BC range from 0.3 mg/kg wet weight (WW) in Manila clams (*Venerupris phillipinarum*) to 3.56 mg/kg WW in Miyagi oysters (*Crassostrea gigas*) [110]. When normalized by a typical portion size of 110 g per portion of uncooked shellfish [52], the resulting concentrations per portion of these shellfish (0.033–0.39 mg/portion) exceeded the highest concentrations per portion in *F*. *distichus* and *N*. *luetkeana* by up to tenfold (Fig 4d). High levels of Cd in cultured British Columbia oysters have led to the loss of some international trade markets [110, 111].

Pb exposure can alter neuromotor and neurosensory function, reduce cognitive function, hemoglobin levels, and kidney function, and cause developmental delays and reproductive impairment [112, 113]. Lead concentrations in our seaweed were only compared to international limits. In the US, the EPA has not assigned an RfD to Pb, but instead relies on the Integrated Exposure Uptake Biokinetic Model for Lead in Children (IEUBK). This Pb blood model is used to predict Pb concentrations in children, considered the most at-risk population. However, seaweed consumption data for children, which is needed to calculate these values, do not exist for our study. The concentrations in three Victoria/Esquimalt Harbour samples (*F*. *distichus* at Rock Bay and Point Hope, and *F*. *spiralis* at Portage Inlet) exceeded both the Chinese and French limits for Pb in seaweed (Table 3, Fig 2e). On a per portion basis, Pb in the two *F*. *distichus* statistical outliers in Victoria Harbour (Rock Bay and Point Hope) also far exceeded concentrations in commonly consumed foods (Fig 4e), but at all other sites they were comparable to concentrations in foods such as fruits, vegetables, and meats.

None of our samples exceeded the RfD-based screening level for methyl Hg (Table 2). Eighteen percent of *F*. *distichus* and 72% of *N*. *luetkeana* samples did exceed the French limit of 0.1 mg/kg (Fig 2f); however, the methodologies for choosing the French limit are not clear and the species of Hg in the French limit was not specified. Additionally, the relative proportions of different Hg species in our samples were not determined. Although most of the Hg in fish and seafood is methyl Hg [114], the proportion of methyl Hg in seaweeds is not well described [9]. Studies that measured total and methyl Hg in seaweed showed methyl Hg below limits of quantitation in *Laminaria digitata* and *Saccharina latissima* [115]; as 1.3 and 3.2 percent of total Hg in *Ascophyllum nodosum* and *Fucus* sp. [116]; and as 1.65% of total Hg in *Fucus* sp. [117]. Hg concentrations in *F*. *distichus* and *N*. *luetkeana* were comparable to those in many commonly consumed foods (Fig 4f). Even the seaweed sample with the highest total Hg concentration (*N*. *luetkeana* from Victoria Jetty) had concentrations per portion lower than those found in marine fish, such as tuna and salmon. Inorganic Hg exposure is of concern because it damages kidneys, stomachs, and intestines in children and adults, and can alter heart function in children when ingested in high amounts [118]. Methyl Hg causes neurologic damage, congenital disease, and immunosuppression, and affects the cardiovascular system [119].

The results of our study identify potential health risks from contaminants when consuming seaweeds harvested from the Salish Sea. The wide ranges of contaminant concentrations among sites suggests that there are certain sites where seaweeds should be avoided, eaten less frequently, or eaten in smaller portions to avoid exceeding CSF- or RfD-based limits, similar to the approach used to set consumption guidelines for fish [120]. As an example of this approach, we used our recalculated consumption rates for Salish Sea *N*. *luetkeana*, *F*. *distichus* and *F*. *spiralis*, which were based on concentrations of Cd, methyl Hg, Pb, BaP, and Σ_40_PCBs measured in this study (Table 4).

This analysis shows that *N*. *luetkeana* can be consumed at all 18 sites at the typical portion size of 5 g DW/person/day for adults without exceeding our calculated screening levels based on USEPA RfDs for Cd or methyl Hg; USEPA CSFs for PCBs or BaP; or the Chinese limit for Pb. On the other hand, *F*. *distichus* can be consumed at an average rate of at least 5 g DW/person/day without exceeding these limits at only 34% of sites. These exceedances are driven primarily by PCBs; at 68% of sites, consuming *F*. *distichus* at 5/g DW/person/day would result in exceeding the CSF for Σ_40_PCBs. Consuming 5 g DW/person/day of *F*. *distichus* from Point Hope would cause the CSF for Σ_40_PCBs and the international limit for Pb to be exceeded and consuming it from Rock Bay in these amounts would cause CSFs for BaP and PCBs and the international limit for Pb to be exceeded. At sites for which only PCBs were problematic, recommended *F*. *distichus* consumption rates could be lowered; these modified consumption rates would range from 2.0 g DW/person/day at Vashon Island to 4.8 g DW/person/day at Hagan Bight. At sites where multiple contaminants exceeded regulated levels, the most conservative recalculated consumption rate could be used. For example, the recommended consumption rate of *F*. *distichus* would then be 1.9 g DW/person/day at Point Hope based on Pb concentrations and 1.2 g DW/person/day at Rock Bay based on BaP concentrations. Using a similar approach for *F*. *spiralis*, the recommended consumption rates would be 4.4 g DW/person/day at Fidalgo Bay based on the Σ_40_PCB concentration and 1.9 g DW/person/day at Portage Inlet based on the Pb concentration. This approach does not account for seasonal variation in contaminant concentrations and would produce different recommendations if international limits were used. Furthermore, it does not apply to children.

For people who rely heavily on Salish Sea seafood in their diets, contaminants in seaweeds need to be considered within the context of total contaminant loads from all seafoods as well as seaweed health benefits and human dimensions (e.g. cultural well-being, sense of place, and availability of local foods for traditional, subsistence, and recreational use) that are not routinely considered in standard risk assessments. While Salish Sea *Fucus* and *Nereocystis* may absorb contaminants, they also contain many chemical constituents that may be beneficial to human health. *Fucus* and *Nereocystis* are high in fiber but low in fat, and contain essential minerals, such as potassium, iron, iodine, zinc, magnesium, and calcium ([73]; Table 3). They are also a source of antioxidants and phytochemicals, such as phlorotannins and fucoidans (Van Alstyne et al., unpublished data.), that have potential human health benefits [5]. Avoiding seaweed consumption because of concerns about contaminants could lead to making less healthy food choices by substituting foods that are low in fiber, high in sugar and unhealthy fats. Furthermore, the substituted foods may have similar contaminant levels (Fig 4). By choosing not to harvest or consume seaweed, people may also lose the benefit of strengthening cultural, family, and community connections and sharing traditional knowledge, in addition to the well-documented physical and psychological benefits of time spent outdoors in nature [121–125]. Thus, although this study focused on risks associated with contaminants in two species of *Fucus* and *N*. *luetkeana* in the Salish Sea, these risks should be considered along with the health benefits that result from consuming these seaweeds.

## 5.0 Conclusions

This study illustrates that concentrations of some chemical contaminants in Salish Sea seaweeds may pose a risk to human health when consumed at the rate specified by the USFDA [52]. PCBs are a concern based on the number of sites that exceeded US-based screening levels for *F*. *distichus* (at 26 out of 38 sites). The only other contaminant that exceeded US-based screening levels was BaP (at 1 out of 38 sites for *F*. *distichus* and 1 out of 3 sites for *F*. *spiralis*). However, when concentrations of all the 162 contaminants we measured in *Fucus* and *Nereocystis* are considered, most were below LOQs or occurred in low concentrations and were below screening levels or international limits (Table 1). In addition to PCBs and BaP, three contaminants (Cd, total Hg, and Pb) exceeded international limits at some sites. One way to retain the potential benefits and reduce risk is to limit the consumption of specific species of seaweed at these sites; therefore, we recalculated consumption rates based on the contaminant concentrations we measured. When compared to commonly consumed foods, concentrations of our six focal contaminants (PCBs, BaP, total As, Cd, total Hg, and Pb) in *F*. *distichus* and *N*. *luetkeana* were generally similar, except for the elevated concentrations of Cd.

PCBs, BaP, As, Cd, Hg and Pb have long been considered contaminants of concern for a wide range of Salish Sea organisms. The spatial pattern of PCBs in seaweed highlights their utility as a biomonitoring tool for identifying sites of concern for ecosystem and human health and as a means to focus remediation efforts to reduce those health concerns. It should be noted that although our study was synoptic and spatially comprehensive, our results were based on single composite samples of each species at each site and included only three of the many edible seaweeds that grow in the Salish Sea. Our results are limited to the chemicals we analyzed and those with USEPA screening levels or other international limits, and do not account for temporal variation, differences between individual blades at a site, seaweed developmental stages or tissue types (e.g., stipes vs. blades), or chemical speciation of metals, which would be particularly important for As because of the high concentrations of total As we measured. Nonetheless, our results point out patterns in seaweed contaminant loads that can inform our use of these resources, and help direct future remediation efforts if used as a biomonitoring tool. Further study is needed to assess the overall safety of seaweeds from the Salish Sea as a food source with consideration of cooking and cleaning techniques; to determine seaweed consumption rates, especially for populations in the region whose diets rely heavily on seafoods; to evaluate the relative benefits and risks of consuming these seafoods; and to ultimately determine whether site-specific guidelines are warranted for their harvest or for siting future seaweed farming facilities.

## Supporting information

S1 Checklist Inclusivity in global research checklist. (DOCX)

S1 Text Calculations of totals for relevant organic chemicals presented in S1 Table. (DOCX)

S2 Text Methods for literature identification presented in S2 Table. (DOCX)

S1 File References cited in supporting information. (DOCX)

S1 Graphical abstract (JPG)

S1 Table Previously published concentrations of Σ_40_PCBs, benzo[*a*]pyrene (BaP), As, Cd, Pb, and Hg in *Fucus* spp. Values for PCBs and BaP are in μg/kg dry weight of seaweed and values for As, Cd, Pb, and Hg are in mg/kg dry weight of seaweed. NS: not specified.
(PDF)

S2 Table Previously published concentrations of As, Cd, Pb, and Hg in *Nereocystis luetkeana*. Values are in mg/kg dry weight of seaweed.
(PDF)

S3 Table Additional information about commonly consumed foods in Fig 4. Concentrations for *F*. *distichus* and *N*. *luetkeana* are in μg/kg DW for PCBs and BaP and in mg/kg DW for total As (tAs), Cd, total Hg, and Pb. In all other foods, they are in μg/kg WW for PCBs and BaP, and in mg/kg WW for total As (tAs), Cd, total Hg, and Pb. Portion sizes for seaweeds are in g DW for *F*. *distichus* and *N*. *luetkeana* and g WW for all other foods.
(PDF)

S4 Table Recommended consumption rates (g DW/day). Based on concentrations of Cd, Hg, Pb, benzo[*a*]pyrene (BaP), the sum of 40 PCB congeners (Σ_40_PCBs), and estimated total PCBs (ET-PCBs) calculated using the method of West et al. (2017) for each site where *Fucus distichus* (FD) and *Fucus spiralis* (FS) were collected. Bolded values are the lowest for a species at a particular site if the lowest value is less than 5 g dry weight/day. Values in parentheses below contaminant types are screening levels based on USEPA CSFs (PCBs and BaP) or RfDs (Cd and methyl Hg). The French limit for Pb was used as USEPA RfDs for Pb have not been assigned. Σ_40_PCB values were based on calculations that used ½ the LOQ when values were less than the LOQ. * Consumption rates that are lower using West et al.’s method [48] for estimating total PCBs relative to the sum of the 40 PCB congeners. ^¥^ Consumption rates that are higher using West et al.’s (2017) method for estimating total PCBs relative to the sum of the 40 PCB congeners. Site codes are as in Fig 1.
(PDF)

S1 Fig Results of estimating total PCBs for *Fucus* spp. at Salish Sea sites from the southernmost sites (left) to the northernmost sites (right) with two different methods. Bars are estimates of summed PCB concentrations (μg^.^kg^-1^ DW) using ½ the LOQ when measurements were below the LOQ: a) calculated by summing the concentrations of all 40 PCB congeners measured, and b) estimating total PCBs using the method of West et al. [48]. Upper ends of the vertical lines are the calculated PCB concentrations when the LOQ was used for concentrations that were less than the LOQ. Lower ends of the bars are calculated values obtained when zero was used for the values less the LOQ. Black dashed horizontal lines indicate the cancer slope factor-based screening level (SL_CSF_). Red bars are concentrations in *Fucus distichus* and yellow hatched bars are concentrations in *Fucus spiralis*. Site codes are as in Fig 1. Site denoted by asterisks are those where the concentration calculated with ½ the LOQ exceeded the screening level using the West et al. method [48], but not the summed method.
(EPS)

## Abbreviations

ARL: acceptable risk limit
BaP: benzo[*a*]pyrene
BW: body weight
CR: consumption rate
CSF: cancer slope factor
DW: dry weight
HCB: hexachlorobenzene
HCH: hexachlorocyclohexane
HPE: heptachlor epoxide
IEUBK: Integrated Exposure Uptake Biokinetic Model
IRIS: Integrated Risk Assessment System
LOQ: limit of quantification
NOAA: National Oceanic and Atmospheric Administration
PAH: polycyclic aromatic hydrocarbon
PBDE: polybrominated diphenyl ether
PCB: polychlorinated biphenyl
PCDD: polychlorinated-dibenzo-*p*-dioxin
PCDF: polychlorinated-dibenzofuran
POP: persistent organic pollutant
RfD: reference dose
SL: screening level
tAS: total arsenic
TCDD: tetrachlorodibenzodioxin
TEF: toxic equivalency factor
TEQ: toxic equivalency
US: United States
USEPA: United States Environmental Protection Agency
USFDA: United States Food and Drug Administration
WDFW: Washington Department of Fish and Wildlife
WHO: World Health Organization
WW: wet weight
Σ_40_PCBs: sum of 40 measured PCBs
