# Supplementary material for: Chemical contaminant levels in edible seaweeds of the Salish Sea and implications for their consumption
Source: PLoS One. 2022 Sep 23;17(9):e0269269. doi: 10.1371/journal.pone.0269269 (PMC9506624; doi:10.1371/journal.pone.0269269)
Supplement: S1 Checklist — (DOCX) [file pone.0269269.s001.docx]

Inclusivity in global research

PLOS’ policy on inclusivity in global research aims to improve transparency in the reporting of research performed outside of researchers’ own country or community and ensures that PLOS publications reporting global research adhere to high standards for research ethics and authorship. Authors of relevant research articles may be asked to complete the questionnaire below, which outlines ethical, cultural, and scientific considerations specific to inclusivity in global research. This questionnaire may be requested when researchers have travelled to a different country to conduct research, if research uses samples collected in another country, research with Indigenous populations or their lands, or if research is on cultural artefacts. Researchers travelling to another country solely to use laboratory equipment will not normally be required to complete the questionnaire. However, the questionnaire can be requested at the journal’s discretion for any submission – if you have been requested to complete this questionnaire by the PLOS journal you submitted to, please do so.

Please complete the questionnaire below and include this as a Supporting Information file with your manuscript. Note that if your paper is accepted for publication, this checklist will be published with your article in the supporting information files. Please ensure that you reference the checklist in the main body of your manuscript. We suggest adding a subsection ‘Inclusivity in global research’ to your Methods section and adding the following sentence: “Additional information regarding the ethical, cultural, and scientific considerations specific to inclusivity in global research is included in the Supporting Information (SX Checklist)”

The questions have been designed to be applicable to a wide range of study types, and there are subsections for both human subjects research and non-human subjects research. If any of the questions are not relevant to your research please mark them as “N/A” as appropriate.

**Ethical considerations, permits and authorship**

*This section is applicable to all research types.*

Provide details as to who granted permissions and/or consent for the study to take place in the Methods section of your manuscript. This should include the names of **all** ethics boards, governmental organizations, community leaders or other bodies that provided approval for the study. If individuals provided approval refer to these people by their role or title but do not list their name(s).

This work is based on JLH’s thesis which the graduate school at Western Washington University, Bellingham, Washington USA approved.

If there were any deviations from the study protocol after approval was obtained please provide details of these changes in the Methods section of your manuscript.

N/A

Did this study involve local collaborators that are residents of the country where the research was conducted or members of the community studied? If you do not have any authors from said communities, please provide an explanation for this below.

Between 2010-2015, JLH was honored to teach seaweed workshops for Tribes in the Salish Sea area which asked her the question that inspired this study: “Is Salish Sea seaweed safe to eat?” In 2015, as a non-Indigenous, graduate school researcher, JLH began this study to answer that question. JLH reached out to her Traditional Food Educator colleagues and Indigenous Healthcare worker colleagues to help guide her in the appropriate approaches and cultural protocols for doing seaweed research in First Nation and Tribe Territories. JLH’s contacts, in turn, reached out to connect her with First Nation Chiefs, healthcare workers, fisheries staff, land managers, and other community members concerned about seaweed contaminants. As well, JLH was connected with the Capitol Regional District (CRD) Harbours and Watershed’s Coordinator who also connected her with Chiefs in the Victoria area and advised her on sampling site selection. JLH is deeply grateful for the generous council of First Nation Chiefs, and Indigenous and non-Indigenous healthcare workers, the CRD staff, and First Nation and non-indigenous scientists who advised her on sample sites, seaweed availability, and important suggestions on how to make research results useful to First Nations and Tribes. However, this article does not have any authors from said communities because they did not meet PLOS’ criteria for authorship.

Everyone listed as an author should meet PLOS’ criteria for authorship and all individuals who meet these criteria should be included in the author byline, rather than the acknowledgements. Authorship criteria is based on the International Committee of Medical Journal Editors (ICMJE) Uniform Requirements for Manuscripts Submitted to Biomedical Journals - for further information please see here: <https://journals.plos.org/plosone/s/authorship>.

**Human subjects research (e.g. health research, medical research, cross-cultural psychology)**

Did you obtain written informed consent from a representative of the local community or region before the research took place? How did you establish who speaks for the community? Details of written informed consent obtained from study participants should be reported separately in the Methods section of your manuscript.

N/A

How did members of the local community provide input on the aims of the research investigation, its methodology, and its anticipated outcome(s)?

N/A

When engaging with the local community, how did you ensure that the informed consent documents and other materials could be understood by local stakeholders?

N/A

Will the findings of the research be made available in an understandable format to stakeholders in the community where the study was conducted (e.g. via a presentation, summary report, copies of publications, etc.)? Please provide details of how this will be achieved.

Yes, finding of the research were made available in an understandable format for each of the 18 Tribes and First Nations in the Salish Sea area who provided access or suggested sampling sites. Findings were shared via email and, if requested, on Zoom with appropriate community members. Those members may have included any of the following: Chief, Council members, healthcare staff, land managers, natural resources staff, fisheries staff, or other concerned community members in the Tribe or First Nation. The email included an on-line link to access the following findings: Summary of Results customized for each Tribe and First Nation (pdf); PowerPoint on Results—also customized (pdf); Seaweed Consumption Rate Tables for 43 sites in the Salish Sea area (pdf); Raw Data on contaminants in seaweed harvested from sites on each Tribes or First Nations reserve lands and/or sites they advised us to sample (Excel); and 2 Scientific Article Drafts: “Chemical Contaminants in Edible Seaweeds of the Salish Sea” (pdf) and “Beneficial Constituents in Salish Sea Seaweeds” (pdf). Due to Covid precautions and restrictions, results presentations were also shared virtually on Zoom or by phone when requested. Further community engagement sharings will be available to Tribes and First Nations upon request from JLH.

**Non-human subjects research using specimens/ animals collected as part of the study, or those housed in archival collections. Examples include archaeology, paleontology, botany and zoology.**

Did the permission you obtained from a local authority to perform the study include an agreement on access to outputs and benefit sharing? This may include procedures to enable fair distribution of the benefits and resources arising from the research performed. Please include any details of Prior Informed Consent and Benefit Sharing Agreements obtained. These may be required by field-specific regulations, for example the Convention on Biological Diversity (CBD) and the associated Nagoya Protocol.

This work did not require a permit from a local authority to perform the study, so we did not have a formal agreement on access to outputs and benefits sharing. JLH did discuss with several Indigenous health workers and First Nation Chiefs how to share results that were useful to Tribes and First Nations. JLH also familiarized herself with the First Nation Health Authority resources for researchers.

If the material used in your study was imported, please A) provide the year it was imported and B) indicate whether permits were obtained to import/export the materials used, C) provide details of any permits obtained. If this information is not available, please indicate this.

A) Seaweed harvested between June 4-Sept 2, 2015 in British Columbia was imported to Washington state. B) No permits were needed to bring seaweed into the US according to the Agriculture Branch Chief, US Customs and Border Protection (CBP), Port of Blaine, Washington. C) N/A

If you used archival specimens, please state how the material used in your study was acquired by the institute it is held in and provide details of any permits obtained for the original excavations/ sample collection. If this information is not available, please indicate this.

N/A

How was the potential cultural significance of the materials collected in your study to local communities considered in your research design? Were Indigenous peoples and/or local researchers and institutions involved with archaeological excavations / collection of specimens? If so, please provide a description of their involvement.

The potential cultural significance of the seaweed collected in this study to local communities was considered in our research design in the following ways: 1) Seaweed is a traditional food for Indigenous people of the Salish Sea area and has been eaten for hundreds, if not thousands of years; 2) As Tribes and First Nations revitalize Indigenous food culture, and interest in seaweed as food and medicine is increasing, there is concern about the levels of contaminants in seaweed and if they pose a health risk; 3) This research was inspired and undertaken to answer a question raised in seaweed workshops sponsored by Tribes and First Nations and taught by JLH: Is Salish Sea seaweed safe to eat, and if so, how much can I eat?

Whenever possible, collection of seaweed specimens involved Indigenous people in Washington, USA and British Columbia, Canada including Tribe or First Nation healthcare workers, fisheries staff, land management staff, or tourism staff, as well as staff who worked for the Tribes or First Nations as biologists or healthcare workers but were non-Indigenous. In Washington, the following assisted with collection of seaweed: Muckleshoot Tribe member drove boat and collected seaweed; Muckleshoot shellfish biologist drove boat and collected seaweed on Tribe-owned land over 3 months; Squaxin Island Tribe members drove boat and collected seaweed; Tulalip Tribes member drove boat and Tulalip Tribes shellfish biologists (non-indigenous) sampled seaweed; Suquamish Tribe Fisheries Director drove boat and assisted at seaweed sample sites; Lower Elwha Klallam Tribe members and healthcare staff collected seaweed. In British Columbia, Stz’uminus First Nation members collected seaweed; Pauquachin First Nation member and two Indigenous health dieticians (indigenous and non-indigenous) collected seaweed; Tsartlip First Nation member oversaw collection of seaweed; T’Sou-ke First Nation fisheries biologist oversaw seaweed collection; Penelakut First Nation member helped collect seaweed and drove boat; Songhees First Nation member helped collect seaweed and Songhees Tourism Coordinator (non-indigenous) helped collect seaweed; Pacheedaht First Nation Referrals Coordinator (non-indigenous) helped collect seaweed.

If your manuscript includes photographs of human remains please indicate whether authors obtained permission from descendants or affiliated cultural communities to do so.

N/A
