# Supplementary material for: Chemical contaminant levels in edible seaweeds of the Salish Sea and implications for their consumption
Source: PLoS One. 2022 Sep 23;17(9):e0269269. doi: 10.1371/journal.pone.0269269 (PMC9506624; doi:10.1371/journal.pone.0269269)
Supplement: S1 Text — (DOCX) [file pone.0269269.s002.docx]

Total chlordanes were calculated as the sum of the concentrations of heptachlor epoxide, oxychlordane, β-chlordane, α-chlordane, *trans*-nonachlor, *cis*-nonachlor, and nonachlor III. Total DDTs were calculated as the sum of the concentrations of 2,2-DDT, 2,4-DDT, and 4,4-DDT. Σ_40_PCBs were calculated as the sums of the concentrations of PCB congeners 17, 18, 28, 31, 33, 44, 49, 52, 66, 70, 74, 82, 87, 95, 99, 101/90, 105, 110, 118, 128, 138/163/164, 149, 151, 153/132, 156, 158, 170, 171, 177, 180, 183, 187/159/182, 191, 194, 195, 199, 205, 206, 208, and 209. Total chrysenes were calculated as the sums of the concentrations of chrysene plus triphenylene; methylated benz[*a*]anthracenes and chrysenes; dimethyl and ethyl-benz[*a*]anthracenes and chrysenes; trimethyl-, methylethyl-, and propyl-benz[*a*]anthracenes and chrysenes; and tetramethyl-, dimethylethyl-, diethyl-, methylpropyl- and butyl-benz[*a*]anthracenes and chrysenes. ΣPBDEs were calculated as the sums of the concentrations of PBDE congeners 28, 47, 49, 66, 85, 99, 100, 153, 154, 155, 183, and four additional PBDE congeners whose congener numbers were not known. To calculate ΣPCDD/F, TEQs were calculated as the sums of the TEQs for 2,3,7,8-tetrachlorodibenzodioxin (TCDD); 1,2,3,7,8-pentachlorodibenzo-*p*-dioxin (PECDD); 1,2,3,4,7,8-hexachlorodibenzo-*p*-dioxin (HXCDD); 1,2,3,6,7,8-HXCDD; 1,2,3,7,8,9-HXCDD; 1,2,3,4,6,7,8-heptachlorodibenzo-*p*-dioxin (HPCDD); octachlorodibenzodioxin (OCDD); 2,3,7,8-tetrachlorodibenzofuran (TCDF); 1,2,3,7,8-pentachlorodibenzofuran (PECDF); 2,3,4,7,8-PECDF; 1,2,3,4,7,8- hexachlorodibenzofuran (HXCDF); 1,2,3,6,7,8-HXCDF; 1,2,3,7,8,9-HXCDF; 2,3,4,6,7,8-HXCDF; 1,2,3,4,6,7,8- heptachlorodibenzofuran (HPCDF); 1,2,3,4,7,8,9-HPCDF; and octachlorodibenzofuran (OCDF), assuming concentrations that were below the limit of quantitation were ½ the LOQ.
