# Supplementary material for: Chemical contaminant levels in edible seaweeds of the Salish Sea and implications for their consumption
Source: PLoS One. 2022 Sep 23;17(9):e0269269. doi: 10.1371/journal.pone.0269269 (PMC9506624; doi:10.1371/journal.pone.0269269)
Supplement: S2 Text — (DOCX) [file pone.0269269.s003.docx]

Literature was identified using Google Scholar and Web of Science, as well as papers referenced in those studies. Key word searches included: “*Fucus distichus*”, “*Fucus gardneri*”, “*Fucus spiralis*”, “*Fucus*”, “*Nereocystis luetkeana*”, “metals”, “cadmium,” “mercury”, “lead”, “PCBs”, “arsenic”, “PAHs”, “BaP”, “POPs”, “macroalgae”, “Phaeophyta”, “seaweed”, and relevant combinations of these terms. Papers were excluded if it was not clear that results were reported as dry weight of tissue or if dry weight calculations could not be made.
