# Supplementary material for: Chemical contaminant levels in edible seaweeds of the Salish Sea and implications for their consumption
Source: PLoS One. 2022 Sep 23;17(9):e0269269. doi: 10.1371/journal.pone.0269269 (PMC9506624; doi:10.1371/journal.pone.0269269)
Supplement: S1 File — (DOCX) [file pone.0269269.s004.docx]

**References cited in supporting information**

1. Almela C, Algora S, Benito V, Clemente M, Devesa V, Súñer M, et al. Heavy metal, total arsenic, and inorganic arsenic contents of algae food products. J Agric Food Chem. 2002; 50:918-923.

2. Barnett BE, Ashcroft CR. Heavy metals in *Fucus vesiculosus* in the Humber Estuary. Environ Poll Ser B Chem Phys. 1985; 9:193-213.

3. Bohn A. Arsenic in marine organisms from West Greenland. Mar Poll Bull. 1975; 6:87-89.

4. Burger, J, Gochfeld M, Jeitner C, Donio M, Pittfield T. Lead (Pb) in biota and perceptions of Pb exposure at a recently designated Superfund beach site in New Jersey. J Toxicol Environ Health Part A. 2012; 75:272-287.

5. Cairrão E, Pereira MJ, Pastorinho MR, Morgado F, Soares AMVM, Guilhermino, L. *Fucus* spp as a mercury contamination bioindicator in coastal areas (Northwestern Portugal). Bull Environ Contamin Toxicol. 2007; 79: 388-395.

6. Caliceti M. Heavy metal contamination in the seaweeds of the Venice Lagoon. Chemosphere. 2002; 47:443-454.

7. Chernova E. The biogeochemical background and trace metal accumulation by brown algae of the genus *Fucus* in coastal waters of the Sea of Japan, the Sea of Okhotsk, and the White Sea. Russ J Mar Biol. 2016; 42:87-96.

8. Desideri D, Cantaluppi C, Ceccotti F, Mell MA, Roselli C, Feduzi L. Essential and toxic elements for human consumption. J Toxic Environ Health. 2016; 79:112-122.

9. Dutton J. Trace metals in the North Sea. Mar Poll Bull. 1973; 4:135-138.

10. Forsberg A, Söderlund S, Petersson R, Pedersen M. Studies of metal content in the brown seaweed, *Fucus vesiculosus*, from the Archipelago of Stockholm. Environ Poll. 1988; 49:245-263.

11. Fuge R, James K. Trace metal concentrations in *Fucus* from the Bristol Channel. Mar Poll Bull. 1974; 5:9-12.

12. García-Salgado S, Quijano M, Bonilla M. Arsenic speciation in edible alga samples by microwave-assisted extraction and high-performance liquid chromatography coupled to atomic fluorescence spectrometry. Analyt Chim Acta. 2012; 714:38-46.

13. García-Seoane R, Aboal JR, Fernández JA. Optimal number of *Fucus vesiculosus* samples to differentiate between sites affected by distinct levels of heavy metal contamination. Aquat Toxicol. 2020; 222:105465. doi.org/10.1016/j.aquatox.2020.105465

14. García-Seoane R, Fernández JA, Boquete MT, Aboal JR. Analysis of intra-thallus and temporal variability of trace elements and nitrogen in *Fucus vesiculosus*: sampling protocol optimization for biomonitoring. J Haz Mat. 2021; 412:125268. doi.org/10.1016/j.jhazmat.2021.125268

15. Giusti L. Heavy metal contamination of brown seaweed and sediments from the UK coastline between the Wear River and the Tees River. Environ Internat. 2001; 26:275-286.

16. Kirso U, Paalme L, Voll M, Urbas E, Irha N. Accumulation of carcinogenic hydrocarbons at the sediment-water interface. Mar Chem. 1990; 30:337-341.

17. Knutzen J, Sortland B. Polycyclic aromatic hydrocarbons (PAH) in some algae and invertebrates from moderately polluted parts of the coast of Norway. Water Res. 1982; 16:421-428.

18. Kreissig KJ, Hansen LT, Jensen PE, Wegeberg S, Geertz-Hansen O, Sloth JJ. Characterisation and chemometric evaluation of 17 elements in ten seaweed species from Greenland. PloS One. 2021; 16:e0243672. doi.org/10.1371/journal.pone.0243672

19. Lai VW, Cullen W, Harrington C, Reimer K. Seasonal changes in arsenic speciation in *Fucus* species. Appl Organometal Chem. 1998; 12:243-251.

20. Lozano G, Hardisson A, Gutierez A, Lafuente M. Lead and cadmium levels in coastal benthic algae (seaweeds) of Tenerife, Canary Islands. Environ Internat. 2003; 28:627-631.

21. Martin M, Nickless G, Stenner R. Concentrations of cadmium, copper, lead, nickel and zinc in the alga *Fucus serratus* in the Severn estuary from 1971 to 1995. Chemosphere. 1997; 34:325-334.

22. Obluchinskaya ED, Pozharitskaya ON, Flisyuk EV, Shikov AN. Formulation, optimization and in vivo evaluation of fucoidan-based cream with anti-inflammatory properties. Marine Drugs. 2021; 19(11), 643. doi:http://dx.doi.org/10.3390/md19110643

23. Pavoni B, Caliceti M, Sperni L, [Sfriso](https://scholar.google.com/citations?user=HuuNosQAAAAJ&hl=en&oi=sra) A. Organic micropollutants (PAHs, PCBs, pesticides) in seaweeds of the macroalgaes of the lagoon in Venice. Ocean Acta. 2003; 26:5-6.

24. Phillips D. Trace metals in the common mussel, *Mytilus edulis* (L), and in the alga *Fucus vesiculosus* (L) from the Region of the Sound (Oresund). Environ Poll. 1979; 18:31-43.

25. Preston A, Jeffries D, Dutton W, Harvey B, Steele A. British Isles coastal waters:the concentrations of selected heavy metals in sea water, suspended matter and biological indicators - a pilot survey. Environ Poll. 1972; 3:69-82.

26. Reis P, Cassiano J, Veiga P, Rubal M, Sousa-Pinto I. *Fucus spiralis* as monitoring tool of metal contamination in the northwest coast of Portugal under the European Water Framework Directives. Environ Monitoring Assess. 2014; 186:5447-5460.

27. Riget F, Johansen P, Asmund G. Natural seasonal variation of cadmium, copper, lead and zinc in brown seaweed (*Fucus vesiculosus*). Mar Poll Bull. 1995; 30:409-413.

28. Riget F, Johansen P, Asmund G. Baseline levels and natural variability of elements in three seaweed species from West Greenland. Mar Poll Bull. 1997; 34:171-176.

29. Sá Monteiro M, Sloth J, Holdt S, Hansen M. Analysis and risk assessment of seaweed. EFSA J. 2019; 17:e170915. doi.org/10.2903/j.efsa.2019.e170915

30. Seelinger U, Edwards P. Concentration factors of copper and lead in seawater and benthic algae. Mar Poll Bull. 1977; 8:16-19.

31. Söderlund S, Forsberg A, Pedersen M. Concentrations of cadmium and other metals in *Fucus vesiculosus* L. and *Fontinalis dalecarlica* Br Eur from the Northern Baltic Sea and the Southern Bothnian Sea. Environ Poll. 1988; 51:197-212.

32. Stenner R, Nickless G. Heavy metals in organisms of the Atlantic coast of SW Spain and Portugal. Mar Poll Bull. 1975; 6:89-92.

33. Struck B, Pelzer R, Ostapczuk P, Emons H, Mohl C. Statistical evaluation of ecosystem properties influencing the uptake of As, Cd, Co, Cu, Hg, Mn, Ni, Pb and Zn in seaweed (*Fucus vesiculosus*) and common mussel (*Mytilus edulis*). Sci Total Environ. 1997; 207:29-42.

34. Taylor V, Jackson B. Concentrations and speciation of arsenic in New England seaweed species harvested for food and agriculture. Chemosphere. 2016; 163:6-13.

35. van Netten C, Hoption Cann S, Morley D, van Netten J. 2000 Elemental and radioactive analysis of commercially available seaweed. Sci Total Environ. 2000; 255:169-175.

36. Varma R, Turner A, Brown M. Bioaccumulation of metals by *Fucus ceranoides* in estuaries of South West England. Mar Poll Bull. 2011; 62:2557-2562.

37. Viana I, Aboal J, Fernández J, Real C, Villares R, Carballeira A. Use of macroalgae stored in an Environmental Specimen Bank for application of some European Framework Directives. Water Res. 2020; 44:1713-1724.

38. Villares R, Carral E, Puente X, Carballeira A. Metal levels in estuarine macrophytes:differences among species. Estuaries. 2005; 28:948-956.

39. Wallenstein FM, Couto RP, Amaral AS, Wilkinson M, Neto AI, Rodrigues AS. Baseline metal concentrations in marine algae from São Miguel (Azores) under different ecological conditions–urban proximity and shallow water hydrothermal activity. Mar Chem. 2008; 87:87-96.

40. Barta E, Branen A, Leung H. Nutritional analysis of Puget Sound Bull Kelp (*Nereocystis*). J Food Sci. 1981; 49:494-497.

41. Whyte JNC, Englar JR. Elemental composition of the marine alga *Nereocystis luetkeana* over the growing season. Can Fish Mar Ser; 1974 Technical Report No.: 509.

42. Whyte JNC, Englar JR, Borgmann PE. [Compositional changes on freshwater leaching of the marine algae *Nereocystis luetkeana* and *Macrocystis integrifolia*](https://www.nrcresearchpress.com/doi/abs/10.1139/f81-025)*.* Can J Fish Aquat Sci. 1981; 38:193-198.

43. Health Canada. Concentrations (pg/g wet wt.) of total PCBs in fatty foods from total diet study in Vancouver. Government of Canada; 2002. Available from: https://www.canada.ca/en/health-canada/services/food-nutrition/food-nutrition-surveillance/canadian-total-diet-study/concentration-contaminants-other-chemicals-food-composites/concentrations-total-pcbs-fatty-foods-total-diet-study-vancouver-2002.html

44. Health Canada. Concentrations (ng/g) of trace elements in 2012 total diet study composites -- sampled from Vancouver. Government of Canada, Bureau of Chemical Safety Food Directorate, Health Products and Food Branch. 2012; Available from: https://www.canada.ca/en/health-canada/services/food-nutrition/food-nutrition-surveillance/canadian-total-diet-study/concentration-contaminants-other-chemicals-food-composites.html

45. Kazerouni N, Sinha R, Che-Han H, Greenberg A, Rothman N. Analysis of 200 food items for benzo[a]pyrene and estimation of its intake in an epidemiologic study. Food Chem Toxicol. 2001; 39:423-436.

46. USFDA (United States Food and Drug Administration). Total diet study: elements results summary statistics market baskets 2006 through 2013. Washington DC: United States Food and Drug Administration, Center for Food Safety and Applied Nutrition, Office of Analytics and Outreach/Exposure Assessment Branch; 2017. Available from: https://www.fda.gov/media/77948/download

47. USFDA (United States Food and Drug Administration). 21CFR Part 101, Section 101.12 Reference amounts customarily consumed per eating occasion. Washington DC: United States Food and Drug Administration. 2018; Available from: https://www.accessdata.fda.gov/scripts/cdrh/cfdocs/cfcfr/CFRSearch.cfm?fr=101.12.

48. West JE, O’Neill SM, Ylitalo GM. Time trends of persistent organic pollutants in benthic and pelagic indicator fishes from Puget Sound, Washington, USA. Envir Contam Toxicol. 2017; 73:207-229.
