# Supplementary figures and images for: Chemical contaminant levels in edible seaweeds of the Salish Sea and implications for their consumption
Source: PLoS One. 2022 Sep 23;17(9):e0269269. doi: 10.1371/journal.pone.0269269 (PMC9506624; doi:10.1371/journal.pone.0269269)

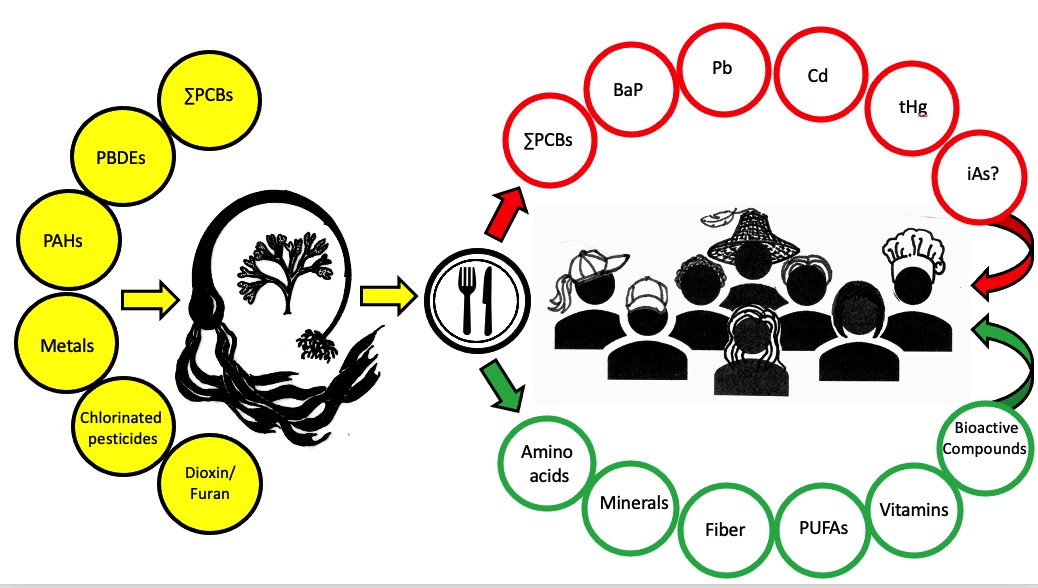

Supplement: S1 Graphical abstract — (JPG) [file pone.0269269.s005.jpg]
