# Supplementary material for: Chemical contaminant levels in edible seaweeds of the Salish Sea and implications for their consumption
Source: PLoS One. 2022 Sep 23;17(9):e0269269. doi: 10.1371/journal.pone.0269269 (PMC9506624; doi:10.1371/journal.pone.0269269)
Supplement: S1 Table — Values for PCBs and BaP are in μg/kg dry weight of seaweed and values for As, Cd, Pb, and Hg are in mg/kg dry weight of seaweed. NS: not specified. (PDF) [file pone.0269269.s006.pdf]

Table S1.

Contaminant	Species	Location	Tissue Type	Mean ± SD (Range)
PCBs (total)	*F. virsoides*	Venice Lagoon, Italy	NS	3.0 ± 1.7 (1.7-4.2) [w]
BaP	*F. serratus*	Norway	thallus except stipe & holdfast	(2-20) [q]
	F. vesiculosus	Gulf of Finland	NS	0.7 [p]
	F. vesiculosus	Norway	thallus except stipe & holdfast	(trace - 64) [q]
Arsenic (inorganic)	*F. spiralis*	New Hampshire, USA	NS	0.04 ± 0.05 [hh]
	F. vesiculosus	Spain	commercial samples	0.34 ± 0.04 [a]
		New Hampshire, USA	NS	0.06 + 0.04 [hh]
		Maine, USA	NS	< detection limit [hh]
Arsenic (total)	*Fucus sp.*	Greenland	NS	26.6 ± 6.6 [r]
	F. ceranoides	SW England	whole thallus	18.8-77.1 [jj]
	F. distichus	Greenland	NS	40.1 ± 5.9 [r]
	*F. distichus**	Vancouver BC, Canada	receptacles (Jul)	8.9 ± 0.5 [s]
		Vancouver BC, Canada	non-reproductive thallus (Jul)	9.0 ± 0.5 [s]
		Vancouver BC, Canada	non-reproductive apices (Feb)	17.1 ± 0.8 [s]
		Vancouver BC, Canada	non-meristematic thallus (Feb)	16.2 ± 0.9 [s]
	F. evanescens	Denmark	NS	14.08 [cc]
	F. serratus	Denmark	NS	30.3-9.58 (105-961) [cc]
	F. spiralis	Denmark	NS	8.94 [cc]
		New Hampshire, USA	NS	16.3 ± 1.4 [hh]
	F. vesiculosus	Spain	commercial samples	50 ± 0.03 [a]
		West Greenland	NS	**35.2-35.8 [c]
		Italy	commercial samples	34.3 ± 6.9 [h]
		France	commercial samples	47 ± 2 [l]
		NW Spain	apices	(28.1-55.6) [m]
		Galicia, NW Spain	dichotomies	(15.9-207) [n]
		Greenland	NS	33.3 ± 7.6 [r]
		West Greenland	apices	25.1-30.7 [bb]
		Denmark	NS	28.4-19.7 (10.4-117) [cc]
		North Sea	NS	46.5 [gg]
		Baltic Sea	NS	21.7 [gg]
		New Hampshire, USA	NS	28.9 ± 2.16 [hh]
		Maine, USA	NS	32.8 ± 3.73 [hh]
		Norway	commercial samples	20 [ii]
	F. virsoides	Venice Lagoon, Italy	whole thallus	40 ± 45 (8-73) [f]
Cadmium	*Fucus* spp.	Barents Sea	whole thallus	1.9 ± 0.5 (1.3-2.7) [g]

	Sea of Okhotsk	whole thallus	3.3 ± 1.9 (1-9) [g]
	Sea of Japan	whole thallus	2.5 ± 1.9 (1-7.8) [g]
	White Sea	whole thallus	0.48 (0.1-1.2) [g]
	Greenland	NS	36.7 ± 4.1 [r]
	UK & Ireland	whole thallus	**1.1-2.5 (0.05-21) [y]
	SW Spain & Portugal	NS	1.7-3.2 [ff]
F. ceranoides	SW England	whole thallus	0.20-1.01 [jj]
	NW Spain	apices	0.19-1.74 [kk]
F. distichus	Greenland	NS	0.95 ± 0.37 [r]
	Murmansk Coast, Russia	whole thallus	0.25-0.88 [v]
F. evanescens	Denmark	NS	0.52 [cc]
F. serratus	Severn Estuary, UK	mature thallus	10.6-35.9 [u]
	Murmansk Coast, Russia	whole thallus	0.31-1.27 [v]
	Denmark	NS	1.04-0.34 (0.63-1.56) [cc]
F. spiralis	Canary Islands	whole thallus	0.62 ± 0.18 [t]
	NW Portugal	soft tissues	0.07-3.58 (0.4-20.8) [z]
	Denmark	NS	0.46 [cc]
	Azores	NS	0.86-1.46 [mm]
F. vesiculosus	Spain	commercial samples	0.55 ± 0.01[a]
	Humber Estuary, England	fronds	5.0-5.2 (1.6-10.5) [b]
	Italy	commercial samples	1.1 ± 0.2 [h]
	NE England	NS	**1.1 (0.5-2.7) [i]
	Stockholm Archipelago	older tissues	4.2-14.7 [j]
	Stockholm Archipelago	apices	6.8-13.3 [j]
	Bristol Channel, England	fronds	3.82-25.6 [k]
	NW Spain	apices	0.47-0.82 [m]
	Galicia, NW Spain	dichotomies	142-5107 [n]
	NE England	top 8-10 cm of thallus	0.02-10.03 [m]
	Greenland	NS	1.42 ± 1.01 [r]
	Öresund Sound, Sweden/Denmark	apices	1.9-3.9 [x]
	Murmansk Coast, Russia	whole thallus	0.30-0.72 [v]
	West Greenland	apices	**3.30 (2.25-4.81) [aa]
	Denmark	NS	0.78-0.37 (0.30-1.97) [cc]
	Bothnian & Baltic Seas, Sweden	older tissues	4.1-17.2 [ee]
	Bothnian & Baltic Seas, Sweden	apices	4.4-12.9 [ee]
	North Sea	NS	0.56 [gg]
	Bothnian & Baltic Seas, Sweden	apices	4.4-12.9 [ee]
	Bothnian & Baltic Seas, Sweden	old thallus	4.1-17.2 [m]
	Norway	commercial samples	0.34 [ii]
	NW Spain	apices	0.59-1.20 [ff]

	F. virsoides	Venice Lagoon, Italy	whole thallus	0.6 $\pm$ 0.3 (0.4-0.8) [f]
Lead	*Fucus* spp.	Greenland	NS	1.59 $\pm$ 2.18 [r]
		SW Spain & Portugal	NS	5-13 [ff]
	F. ceranoides	SW England	whole thallus	2.93-14.3 [jj]
		NW Spain	apices	0.38-7.45 [kk]
		Galicia, Spain	whole thallus	4.40 [ll]
	F. distichus	Greenland	NS	0.24 $\pm$ 0.07 [r]
		Murmansk Coast, Russia	whole thallus	0.31-0.97 [v]
	F. evanescens	Denmark	NS	0.51 [cc]
	F. gardneri	Raritan Bay, NJ, USA	NS	0.22-230 [d]
	F. serratus	Severn Estuary, UK	mature thallus	6.4-7.9 [u]
		Murmansk Coast, Russia	whole thallus	0.13-1.05 [v]
		Denmark	NS	0.47-0.18 (0.24-0.87) [cc]
	F. spiralis	Canary Islands	whole thallus	4.96 $\pm$ 2.44 [t]
		Denmark	NS	0.96 [cc]
		Galicia, Spain	whole thallus	5.71 [ll]
		Azores	NS	0.16-0.38 [mm]
	F. vesiculosus	Spain	commercial samples	0.51 $\pm$ 0.04 [a]
		Humber Estuary, England	fronds	5-8.1 (2.7-21.5) [b]
		Italy	commercial samples	4 $\pm$ 0.3 [h]
		NE England	NS	**4.5 (3.1-10) [i]
		Stockholm Archipelago	older tissues	0.9-6.9 [j]
		Stockholm Archipelago	apices	1.7-6.6 [j]
		NW Spain	apices	0.25-1.62 [m]
		Galicia, NW Spain	dichotomies	0.18-1.68 [n]
		NE England	upper 8-10 cm of fronds	0.1-12.1 [o]
		Greenland	NS	0.10 $\pm$ 0.06 [r]
		Öresund Sound, Sweden/Denmark	apices	14.0-27.4 [x]
		Ireland & UK	whole thallus	2.1-4.0 (0.5-9.0) [y]
		West Greenland	apices	**0.25 (0.047-0.70) [aa]
		Murmansk Coast, Russia	whole thallus	0.12-0.77 [v]
		Raritan Bay, New York, USA	NS	(1.3-2.4) [dd]
		Bothnian & Baltic Seas, Sweden	older tissues	2.1-7.2 [ee]
		Bothnian & Baltic Seas, Sweden	apices	2.0-11.7 [ee]
		North Sea	NS	1.86 [gg]
		Baltic Sea	NS	1.04 [gg]
		Denmark	NS	0.90-1.73 (0.19-9.60) [cc]
		Norway	commercial samples	0.38 [ii]
		NW Spain	apices	0.35-3.56 [kk]
	F. virsoides	Venice Lagoon, Italy	whole thallus	1.6 $\pm$ 0.6 (1.2-2.0) [f]

Mercury	*Fucus* spp.	NW Portugal	receptacles	(0.012-0.061) [e]
		Greenland	NS	<0.023 [r]
	F. distichus	Greenland	NS	<0.023 [r]
	F. evanescens	Denmark	NS	0.008 [cc]
	F. gardneri	Raritan Bay, NJ, USA	NA	0.0068 [d]
	F. ceranoides	NW Spain	apices	0.020-0.027 [kk]
	F. serratus	Denmark	NS	0.009-0.003 (0.005-0.015) [cc]
	F. spiralis	Denmark	NS	0.019 [cc]
	F. vesiculosus	NW Spain	commercial samples	0.036 $\pm$ 0.006 [a]
		NW Spain	apices	0.015-0.056 [m]
		Galicia, NW Spain	dichotomies	0.004-0.060 [n]
		Greenland	NS	<0.023 [r]
		North Sea	NS	0.010 [gg]
		Baltic Sea	NS	0.0018 [gg]
		Denmark	NS	0.012-0.007 (0.003-0.042) [cc]
		Norway	commercial samples	1.08 [ii]
		NW Spain	apices	0.020 – 0.031 [kk]

*Reported as *F. gardneri*
** Reported as a geometric mean

[a][1]; [b] [2]; [c] [3]; [d] [4]; [e] [5]; [f] [6]; [g] [7]; [h] [8]; [i] [9]; [j] [10]; [k] [11]; [l] [12]; [m] [13]; [n][14]; [o] [15]; [p] [16]; [q] [17]; [r] [18]; [s] [19]; [t] [20]; [u] [21]; [v] [22]; [w] [23]; [x] [24]; [y] [25]; [z] [26]; [aa] [27]; [bb] [28]; [cc][29]; [dd] [30]; [ee] [31]; [ff] [32]; [gg] [33]; [hh] [34]; [ii] [35]; [jj] [36]; [kk] [37]; [ll] [38]; [mm] [39]
