# Supplementary material for: Chemical contaminant levels in edible seaweeds of the Salish Sea and implications for their consumption
Source: PLoS One. 2022 Sep 23;17(9):e0269269. doi: 10.1371/journal.pone.0269269 (PMC9506624; doi:10.1371/journal.pone.0269269)
Supplement: S2 Table — Values are in mg/kg dry weight of seaweed. (PDF) [file pone.0269269.s007.pdf]

Table S2.

Contaminant	Location	Tissue Type	Mean $\pm$ SD (Range)
Arsenic (inorganic)	Vancouver, BC, Canada	fronds	2.7 [d]
	Vancouver, BC, Canada	stipes	0.5 [d]
Arsenic (total)	Vancouver, BC, Canada	fronds	92.4 [d]
	Vancouver, BC, Canada	stipes	48.8 [d]
	Bamfield, BC, Canada	commercial samples	(66 -79) [a]
	Washington, USA	fronds	62 $\pm$ 1.4 [b]
	Washington, USA	stipes	72 $\pm$ 1.3 [b]
	Washington, USA	bulbs	41 $\pm$ 1.0 [b]
Cadmium	Vancouver, BC, Canada	fronds	2 [c]
	Bamfield, BC, Canada	commercial samples	0.30 - 2.76 [a]
Lead	Bamfield, BC, Canada	commercial samples	<0.01-0.08 [a]
	Vancouver, BC, Canada	fronds	0.9 [c]
Mercury	Bamfield, BC, Canada	commercial samples	Not detected (<0.05) [a]
	Vancouver, BC, Canada	fronds	Not detected (<0.05) [c]

[a] [35]; [b] [40]; [c] [41]; [d] [42]
