# Supplementary material for: Chemical contaminant levels in edible seaweeds of the Salish Sea and implications for their consumption
Source: PLoS One. 2022 Sep 23;17(9):e0269269. doi: 10.1371/journal.pone.0269269 (PMC9506624; doi:10.1371/journal.pone.0269269)
Supplement: S3 Table — Concentrations for F. distichus and N. luetkeana are in μg/kg DW for PCBs and BaP and in mg/kg DW for total As (tAs), Cd, total Hg, and Pb. In all other foods, they are in μg/kg WW for PCBs and BaP, and in mg/kg WW for total As (tAs), Cd, total Hg, and Pb. Portion sizes for seaweeds are in g DW for F. distichus and N. luetkeana and g WW for all other foods. (PDF) [file pone.0269269.s008.pdf]

Table S3.

Analyte	Fig 3 Label	Description	Concen-tration	Portion Size (g)
PCBs	Mayonnaise	Mayonnaise	0.001 [a]	15 [e]
PCBs	Chocolate	Chocolate bars	0.24 [a]	30 [e]
PCBs	French fries	French fries	0.1118 [a]	70 [e]
PCBs	Hot dog	Hot dog	0.277 [a]	55 [e]
PCBs	Cheddar cheese	Cheddar cheese	0.41 [a]	30 [e]
PCBs	Butter	Butter	0.84 [a]	15 [e]
PCBs	Eggs	Eggs	0.36 [a]	50 [e]
PCBs	Shellfish	Shellfish	0.25 [a]	85 [e]
PCBs	Pizza	Pizza	0.16 [a]	140 [e]
PCBs	*F. distichus*	*F. distichus*: mean of all sites except Smith Cove, Vashon Island (June), and Rock Bay	10.02 [f]	5 [e]
PCBs	*F. distichus* - SC	*F. distichus* collected from Smith Cove	18.9 [f]	5 [e]
PCBs	*F. distichus* - VI6	*F. distichus* collected from Vashon Island (June)	19.6 [f]	5 [e]
PCBs	*F. distichus* - RB	*F. distichus* collected from Rock Bay	21.2 [f]	5 [e]
PCBs	Marine fish	Marine fish	4.2 [a]	85 [e]
PCBs	Freshwater fish	Freshwater fish	7.2 [a]	85 [e]
BaP	Bacon	Bacon, pan-fried, well done	0.2 [c]	16 [c]
BaP	Hotdog	Hot dogs, grilled, very well done	0.05 [c]	55 [e]
BaP	*F. distichus*	*F. distichus*: mean of all sites except Rock Bay	1.14 [6]	5 [e]
BaP	Fried fish	Fish sandwich, deep fried, fast food	0.07 [c]	112 [c]
BaP	Spinach	Spinach, fresh and frozen	0.10 [c]	85 [e]
BaP	Smoked fish	Smoked fish	0.10 [c]	55 [e]
BaP	Pretzels	Pretzels	0.37 [c]	30 [c]
BaP	Broccoli	Broccoli	0.17 [c]	85 [e]
BaP	Potato	White potato	0.17 [c]	110 [e]
BaP	Popcorn	Popped popcorn	0.56 [c]	30 [c]
BaP	Yogurt	Yogurt, flavored/frozen	0.18 [c]	227 [c]
BaP	Kale	Kale	0.47 [c]	85 [e]
BaP	Pumpkin pie	Pumpkin pie	0.47 [c]	135 [c]
BaP	Hamburger	Hamburger, grilled/BBQ, very well done	1.520 [c]	85 [c]
BaP	*F. distichus*-RB	*F. distichus* collected from Rock Bay	64.63 [f]	5 [f]
BaP	Chicken	Chicken with bone/skin, grilled/BBQ, well done	4.570 [c]	96 [c]
BaP	Steak	Steak, grilled/BBQ, very well done	4.860 [c]	112 [c]
tAs	Peanut butter	Peanut butter	0.021 [b]	32 [e]
tAs	Bread	Bread, whole wheat	0.018 [b]	50 [e]
tAs	Raspberries	Raspberries	0.013 [b]	140 [e]
tAs	Mushrooms	Mushrooms	0.028 [b]	85 [e]
tAs	Cereal	Cereal, wheat, rice, bran	0.076 [b]	40 [e]
tAs	Hamburger	Hamburger	0.047 [b]	85 [e]
tAs	Rice	Rice	0.064 [b]	140 [e]
tAs	Freshwater fish	Fish, fresh water	0.357 [b]	85 [e]
tAs	Shellfish	Shellfish	0.362 [b]	85 [e]
tAs	Canned fish	Fish, canned	0.752 [b]	85 [e]
tAs	*F. distichus*	*F. distichus*: mean of all sites except Vashon Island (August)	26.50 [f]	5 [e]
tAs	*F. distichus*-VI8	*F. distichus* collected from Vashon Island (August)	37.01 [f]	5 [e]
tAs	*N. luetkeana*	*N. luetkeana*: mean of all sites except Brochie Rocks	70.89 [f]	5 [e]
tAs	Marine fish	Fish, marine	5.529 [b]	85 [e]
tAs	*N. luetkeana* - BR	*N. luetkeana* collected from Brochie Rocks	98.92 [f]	5 [e]
Cd	Marine fish	Fish, marine	0.004 [b]	85 [e]
Cd	Chocolate	Chocolate bars	0.011 [b]	30 [e]
Cd	Whole wheat bread	Bread, whole wheat	0.020 [b]	50 [e]
Cd	Cereal	Cereal, wheat, rice, bran	0.038 [b]	40 [e]

Cd	Strawberries	Strawberries	0.011 [b]	140 [e]
Cd	Celery	Celery	0.027 [b]	85 [e]
Cd	Nuts	Nuts	0.084 [b]	30 [e]
Cd	Carrots	Carrots	0.030 [b]	85 [e]
Cd	Potato chips	Potato chips	0.118 [b]	30 [e]
Cd	Pasta	Pasta, plain	0.026 [b]	140 [e]
Cd	Potatos	Potatos, baked with skins	0.057 [b]	70 [e]
Cd	Lettuce	Lettuce	0.082 [b]	85 [e]
Cd	Spinach	Spinach	0.114 [b]	85 [e]
Cd	Sunflower seeds	Sunflower Seeds (shelled), roasted, salted	0.389 [d]	30 [e]
Cd	*F. distichus*	*F. distichus*: mean of all sites except Jefferson Beach	2.480 [f]	5 [e]
Cd	*F. distichus* - JB	*F. distichus* collected from Jefferson Beach	4.250 [f]	5 [e]
Cd	*N. luetkeana*	*N. luetkeana*: mean of all sites	5.691 [f]	5 [e]
Pb	Hot dogs	Hot dogs	0.004 [b]	55 [e]
Pb	Marine fish	Marine fish	0.003 [b]	85 [e]
Pb	French fries	French fries	0.004 [b]	70 [e]
Pb	Chocolate	Chocolate bars	0.009 [b]	30 [e]
Pb	*N. luetkeana*	*N. luetkeana*: mean of all sites except Four-Mile Rock and Victoria Jetty	0.060 [f]	5 [e]
Pb	Pizza	Pizza	0.002 [b]	140 [e]
Pb	*F. distichus*	*F. distichus*: mean of all sites except Rock Bay and Point Hope	0.091 [f]	5 [e]
Pb	Beets	Beets	0.007 [b]	85 [e]
Pb	Broccoli	Broccoli	0.007 [b]	85 [e]
Pb	*N. luetkeana* - FM	*N. luetkeana* collected from Four-Mile Rock	0.132 [f]	5 [e]
Pb	*N. luetkeana* - VJ	*N. luetkeana* collected from Victoria Jetty	0.136 [f]	5 [e]
Pb	Spinach	Spinach	0.009 [b]	85 [e]
Pb	Rice	Rice	0.007 [b]	140 [e]
Pb	Shellfish	Shellfish	0.016 [b]	85 [e]
Pb	Apricots	Apricots	0.027 [b]	140 [e]
Pb	*F. distichus* - RB	*F. distichus* collected from Rock Bay	9.706 [b]	5 [e]
Pb	*F. distichus* - PH	*F. distichus* collected from Point Hope	13.192 [b]	5 [e]
Hg	Peanut butter	Peanut butter, smooth/creamy	0.0002 [d]	32 [e]
Hg	Eggs	Eggs, boiled	0.0001 [d]	50 [e]
Hg	Bread	Bread, white, enriched	0.0001 [d]	50 [e]
Hg	Spinach	Spinach, fresh/frozen, boiled	0.0003 [d]	85 [e]
Hg	Rice	Rice, white, enriched, cooked	0.0004 [d]	140 [e]
Hg	Clam chowder	Clam chowder, New England -- canned, condensed, prepared with whole milk.	0.0003 [d]	245 [e]
Hg	Mushrooms	Mushrooms, raw	0.001 [d]	85 [e]
Hg	Catfish	Catfish, pan-cooked with oil	0.003 [d]	85 [e]
Hg	*F. distichus*	*F. distichus*: mean of all sites except Wing Point and Deep Bay	0.058 [f]	5 [e]
Hg	Fish sticks	Fish stick or patty, frozen, oven-cooked	0.004 [d]	85 [e]
Hg	Shrimp	Shrimp, boiled	0.006 [d]	85 [e]
Hg	*N. luetkeana*	*N. luetkeana*: mean of all sites except Victoria Jetty	0.116 [f]	5 [e]
Hg	*F. distichus*-WP	*F. distichus* collected from Wing Point	0.192 [f]	5 [e]
Hg	*F. distichus*-DB	*F. distichus* collected from Deep Bay	0.205 [f]	5 [e]
Hg	*N. luetkeana* -VJ	*N. luetkeana* collected from Victoria Jetty	0.250 [f]	5 [e]
Hg	Tuna noodle casserole	Tuna noodle casserole, homemade	0.016 [d]	140 [e]
Hg	Salmon	Salmon, steaks/fillets baked	0.021 [d]	85 [e]
Hg	Tuna	Tuna, canned in water, drained	0.136 [d]	85 [e]

[a][43]; [b][44]; [c][45]; [d] 46]; [e] [47]; and [f] this study.
