# Supplementary material for: Chemical contaminant levels in edible seaweeds of the Salish Sea and implications for their consumption
Source: PLoS One. 2022 Sep 23;17(9):e0269269. doi: 10.1371/journal.pone.0269269 (PMC9506624; doi:10.1371/journal.pone.0269269)
Supplement: S4 Table — Based on concentrations of Cd, Hg, Pb, benzo[a]pyrene (BaP), the sum of 40 PCB congeners (Σ40PCBs), and estimated total PCBs (ET-PCBs) calculated using the method of West et al. (2017) for each site where Fucus distichus (FD) and Fucus spiralis (FS) were collected. Bolded values are the lowest for a species at a particular site if the lowest value is less than 5 g dry weight/day. Values in parentheses below contaminant types are screening levels based on USEPA CSFs (PCBs and BaP) or RfDs (Cd and methyl Hg). The French limit for Pb was used as USEPA RfDs for Pb have not been assigned. Σ40PCB values were based on calculations that used ½ the LOQ when values were less than the LOQ. * Consumption rates that are lower using West et al.’s method [48] for estimating total PCBs relative to the sum of the 40 PCB congeners. ¥ Consumption rates that are higher using West et al.’s (2017) method for estimating total PCBs relative to the sum of the 40 PCB congeners. Site codes are as in Fig 1. (PDF) [file pone.0269269.s009.pdf]

Table S4.

Collection Site	Species	*Collection Date*	*Cd (16)*	*Hg (1.6)*	*Pb (5)*	*BaP (16)*	*Σ_{40}PCBs (8)*	*ET-PCBs (8)*
AB	FD	08/17/15	≥5	≥5	≥5	≥5	≥5	**4.9***
AS	FD	09/03/15	≥5	≥5	≥5	≥5	3.7	**3.6***
BB	FD	07/04/15	≥5	≥5	≥5	≥5	≥5	≥5
BM	FD	08/31/15	≥5	≥5	≥5	≥5	2.7	**2.6***
BR	FD	08/07/15	≥5	≥5	≥5	≥5	4.5	**4.3***
CA	FD	09/02/15	≥5	≥5	≥5	≥5	**3.7**	3.8¥
CB	FD	06/04/15	≥5	≥5	≥5	≥5	≥5	**4.4***
CH	FD	07/07/15	≥5	≥5	≥5	≥5	3.9	**3.8***
CI	FD	06/05/15	≥5	≥5	≥5	≥5	**4.2**	4.3¥
CM	FD	08/05/15	≥5	≥5	≥5	≥5	≥5	≥5
DB	FD	08/13/15	≥5	≥5	≥5	≥5	≥5	≥5
DP	FS	09/02/15	≥5	≥5	≥5	≥5	≥5	≥5
EF	FD	09/01/15	≥5	≥5	≥5	≥5	4.9	**4.4***
EH	FD	08/07/15	≥5	≥5	≥5	≥5	4.0	**3.9***
ES	FD	08/16/15	≥5	≥5	≥5	≥5	≥5	≥5
FB	FS	08/26/15	≥5	≥5	≥5	≥5	4.4	**4.3***
FM	FD	07/01/15	≥5	≥5	≥5	≥5	≥5	4.6*
FR	FD	08/02/15	≥5	≥5	≥5	≥5	4.8	**4.3***
FW	FD	07/03/15	≥5	≥5	≥5	≥5	3.7	**3.2***
GM	FD	08/10/15	≥5	≥5	≥5	≥5	≥5	**5.0***
HB	FD	07/30/15	≥5	≥5	≥5	≥5	4.8	**4.4***
JB	FD	06/30/15	≥5	≥5	≥5	≥5	≥5	≥5
KB	FD	08/12/15	≥5	≥5	≥5	≥5	≥5	≥5
LT	FD	08/25/15	≥5	≥5	≥5	≥5	3.6	**3.8¥**
MF	FD	06/16/15	≥5	≥5	≥5	≥5	5.0	**4.8***
PA	FD	07/17/15	≥5	≥5	≥5	≥5	4.4	**4.1***
PE	FD	08/27/15	≥5	≥5	≥5	≥5	≥5	≥5
PH	FD	07/18/15	≥5	≥5	**1.9**	≥5	3.1	2.7*
PI	FD	07/05/15	≥5	≥5	≥5	≥5	≥5	≥5
PO	FS	08/03/15	≥5	≥5	**1.9**	3.8	2.8	**2.4***
PP	FD	07/29/15	≥5	≥5	≥5	≥5	4.4	**3.8***
RB	FD	07/18/15	≥5	≥5	2.6	**1.2**	1.9	1.6*
RW	FD	09/06/15	≥5	≥5	≥5	≥5	3.3	**2.6***
SB	FD	08/09/15	≥5	≥5	≥5	≥5	**3.7**	**3.7**
SC	FD	06/02/15	≥5	≥5	≥5	≥5	2.1	**2.0***
SI	FD	07/30/15	≥5	≥5	≥5	≥5	3.3	**3.7¥**
SN	FD	08/04/15	≥5	≥5	≥5	≥5	2.8	**2.6**
TJ	FD	08/10/15	≥5	≥5	≥5	≥5	2.4	**2.2***
VI	FD	07/02/15	≥5	≥5	≥5	≥5	3.3	**2.8***

VI	FD	*08/14/15*	*≥5*	*≥5*	*≥5*	*≥5*	***2.9***	***2.9***
VI	FD	*06/02/15*	*≥5*	*≥5*	*≥5*	*≥5*	*2.0*	***2.1***[¥]
VJ	FD	*07/18/15*	*≥5*	*≥5*	*≥5*	*≥5*	*2.1*	***2.2***[¥]
WP	FD	*06/30/15*	*≥5*	*≥5*	*≥5*	*≥5*	*2.6*	***2.8***[¥]
